# Digital Interventions for Symptoms of Borderline Personality Disorder: Systematic Review and Meta-Analysis

**DOI:** 10.2196/54941

**Published:** 2024-11-29

**Authors:** Julia A B Lindsay, Niall M McGowan, Thomas Henning, Eli Harriss, Kate E A Saunders

**Affiliations:** 1 Department of Psychiatry University of Oxford Oxford United Kingdom; 2 School of Medicine and Biomedical Sciences University of Oxford Oxford United Kingdom; 3 Bodleian Health Care Libraries University of Oxford Oxford United Kingdom; 4 Oxford Health NHS Foundation Trust Warneford Hospital Oxford United Kingdom

**Keywords:** borderline personality disorder, BPD, digital intervention, digital health, digital therapeutics, persuasive system design, systematic review, meta-analysis, suicidal ideation, paranoia, single symptom, mental health, behavior change, treatment, effectiveness, symptom, suicide, mobile phone

## Abstract

**Background:**

Borderline personality disorder (BPD) is a mental health condition with insufficient care availability worldwide. Digital mental health interventions could reduce this treatment gap. Persuasive system design (PSD) is a conceptual framework outlining elements of digital interventions that support behavior change.

**Objective:**

This systematic review aims to characterize digital interventions targeting BPD symptoms, assess treatment efficacy, and identify its association with intervention features, including PSD elements.

**Methods:**

A systematic review of automated digital interventions targeting symptoms of BPD was conducted. Eligible studies recruited participants aged ≥18 years, based on a diagnosis of BPD or one of its common comorbidities, or as healthy volunteers. OVID Embase, OVID MEDLINE, OVID PsycINFO, and the Cochrane Central Register for Controlled Trials were searched on July 19, 2022, and February 28, 2023. Intervention characteristics were tabulated. A meta-analysis of randomized controlled trials (RCTs) determined treatment effects separately for each core symptom of BPD using Hedges *g*. Associations between the treatment effect and intervention features, including PSD elements, were assessed by subgroup analysis (Cochran *Q* test). Risk of bias was assessed using the Cochrane Risk of Bias 2 tool for RCTs and the National Institutes of Health Quality Assessment Tool for pre-post studies.

**Results:**

A total of 40 (0.47%) publications out of 8520 met the inclusion criteria of this review, representing 6611 participants. Studies comprised examinations of 38 unique interventions, of which 32 (84%) were RCTs. Synthesis found that included interventions had the following transdiagnostic treatment targets: severity of BPD symptoms (4/38, 11%), suicidal ideation (17/38, 45%), paranoia (5/38, 13%), nonsuicidal self-injury (5/38, 13%), emotion regulation (4/38, 11%), and anger (3/38, 8%). Common therapeutic approaches were based on dialectical behavioral therapy (8/38, 21%), cognitive behavioral therapy (6/38, 16%), or both (5/38, 13%). Meta-analysis found significant effects of digital intervention for both symptoms of paranoia (Hedges *g*=–0.52, 95% CI –0.86 to –0.18; *P*=.01) and suicidal ideation (Hedges *g*=–0.13, 95% CI –0.25 to –0.01; *P*=.03) but not overall BPD symptom severity (Hedges *g*=–0.17, 95% CI –0.42 to 0.10; *P*=.72). Subgroup analysis of suicidal ideation interventions found that evidence-based treatments such as cognitive behavioral therapy and dialectical behavior therapy were significantly more effective than alternative modalities (Cochran *Q*=4.87; *P*=.03). The degree of human support was not associated with the treatment effect. Interventions targeting suicidal ideation that used reminders, offered self-monitoring, and encouraged users to rehearse behaviors were associated with a greater reduction in ideation severity.

**Conclusions:**

Evidence suggests that digital interventions may reduce the symptoms of suicidal ideation and paranoia and that the design of digital interventions may impact the efficacy of treatments targeting suicidal ideation. These results support the use of transdiagnostic digital interventions for paranoia and suicidal ideation.

**Trial Registration:**

PROSPERO CRD42022358270; https://tinyurl.com/3mz7uc7k

## Introduction

### Borderline Personality Disorder

Borderline personality disorder (BPD) is a mental health condition characterized by 9 core symptoms usually involving intense and volatile emotional reactivity, identity disturbance, and interpersonal difficulties; however, symptom presentation is highly heterogeneous [1]. People seeking treatment for BPD represent a significant proportion of those referred to psychiatric clinical services: about 22% of inpatients and 12% of outpatients globally [2]. The demand for BPD treatment vastly outstrips availability, with nearly 6000 people seeking treatment for the condition per evidence-based care provider in the United States. Furthermore, the cost per patient is estimated to be more than double that of patients with depression [3,4].

This treatment gap could be reduced by digital interventions, which inform users and support behavior changes to reduce symptom severity and functional impairment [5,6]. Although few digital interventions are specifically designed for BPD, many address transdiagnostic symptoms, making them potentially effective for BPD treatment due to the disorder’s diverse symptom presentation. For example, many digital interventions have a treatment target of emotion regulation, a core symptom of BPD that is also common in mood, substance use, and eating disorders [7]. People with BPD tend to use less effective emotion regulation strategies than healthy controls [8]. Poor emotion regulation is associated with behavioral control and drives maladaptive behaviors such as impulsive anger and nonsuicidal self-injury (NSSI), which are also symptoms of BPD [9-11]. Paranoia is another transdiagnostic symptom that can be treated effectively, although few interventions focus specifically on paranoia in BPD [12]. Symptom-based care offers treatment without focusing on a specific diagnosis, which could help reduce pathologization—or the unnecessary labeling of illness—in individuals diagnosed with BPD, many of whom are opposed to the label of personality disorder [13]. Digital interventions offer advantages such as access without diagnosis, lower cost, greater anonymity, and continuous availability. Despite concerns about limited clinician contact, self-guided digital interventions can be as effective as traditional face-to-face alternatives [14]. However, several challenges remain, such as nonadherence, limited assessment of efficacy in controlled trials, and poor user experience [15,16].

### Persuasive System Design

The importance of user experience in digital health has been demonstrated by Kelders et al [17], who showed that persuasive system design (PSD) was associated with increased adherence to digital interventions. The concept of PSD proposes that user experience and interface elements, detailed in Tables 1 and 2, support behavior change in users [18]. For example, reminders to engage with the intervention and opportunities to rehearse new skills may help users reach their treatment goals by maintaining their adherence to behavioral and cognitive interventions. Research on adherence typically compares characteristics between adherers and nonadherers, thereby putting the burden of adherence onto the user [19]. However, given the evidence for PSD, adherence may be a product of the interaction between the user and the interface rather than a reflection of the users’ characteristics.

**Table 1:** Coding scheme for the persuasive system design elements that offer primary task support^a^.

Primary task support	Concept	Implementation example
Reduction	Breaking goals down into smaller tasks, for example, skills modules, makes them easier to achieve	DBT^b^ Coach [20]: presents the user with a series of tasks depending on the DBT skill being practiced in each session
Tunneling	Guiding the user through tasks in a predefined order reduces the mental burden of learning by making it obvious what should be done next	Life Buoy [21]: the user must complete each module in the sequence to unlock the next
Tailoring	The interventions’ content and user interface is adapted to suit different groups of users. This content-matching means users receive more relevant information or guidance to help them reach their goals	Man Therapy [22]: external resources signposted depend on the US state in which the user is located
Personalization	The interventions’ content and user interface is adapted for each user or provides options that users may choose between. Personalization may increase engagement by helping the user feel seen	Be a Mom [23]: intervention uses the names of the user (a mother) and her baby
Self-monitoring	Providing users with either visual indicators of their progress or opportunities for self-reflecting on progress supports their motivation to reach their goals	mDiary [24]: 10 mood-related variables are logged daily, and charts showing change over time are presented to the user
Simulation	Estimating the cause and effect of different behavioral trajectories can motivate users to change or maintain their behavior	Sleep Scholar [25]: shares information about the consequences of poor sleep strategies
Rehearsal	Practicing skills in a comfortable environment builds the habit of using them as needed	Johnson et al [26]: Users are encouraged to practice relaxation skills and create an implementation intention to use their skills

^a^The scheme and table are adapted from the study by Kelders et al [17].

^b^DBT: dialectical behavior therapy.

**Table 2:** Coding scheme for the persuasive system design elements that offer dialogue support^a^.

Dialogue support	Concept	Implementation example
Rewards	Incentivizes engagement with the intervention. In this analysis, participant compensation is counted as a reward	TEC^b^ [27]: awards points for each TEC trial that the user completes. In the trial, participant compensation was based on the number of points earned
Reminders	Prompting users may increase the likelihood of their sustained engagement with the intervention and prevent forgetful nonadherence. Reminders could be related to intervention use or the target behavior	priovi [28]: users can choose to register for daily e-mails and SMS text messages
Suggestion	Describing and endorsing target behavior may encourage change in users	Bernstein et al [29]: throughout the day, the user is prompted to use emotion regulation skills toward any “current distressing emotions”
Similarity	Users may resonate more with interventions that feature people like the users or environments like their own	FitMindKit [30]: modules are presented by characters who have the same mental health challenges as the user
Liking	Appealing and cohesive user interfaces may promote engagement with the intervention	Life Buoy [21]: features a sailing-themed interface in which each module is represented by an island. The interface was designed in consultation with a lived-experience group
Social role	Interventions that take on a familiar anthropomorphic role (eg, coach, instructor, or buddy) may be more naturally adopted by users	FitMindKit [30]: features an expert narrator who takes on the role of the instructor

^a^The scheme and table are adapted from the study by Kelders et al [17].

^b^TEC: therapeutic evaluative conditioning.

The proposed mechanism of action is that PSD elements increase user adherence, which in turn supports greater treatment effect. Different elements have different effects: some may reduce goal behaviors into smaller, manageable changes, while others promote frequent use or lend increased credibility to the intervention [17]. Accordingly, this review aims to (1) identify and describe the therapeutic approach, duration, intended frequency of use, and user interface of digital interventions treating BPD symptoms in community and secondary care settings; (2) conduct a meta-analysis to determine the efficacy of digital interventions compared to active or passive control groups; and (3) determine using subgroup analysis whether there were associations between treatment efficacy and characteristics of the intervention, such as the therapeutic modality, the degree of human support, and the use of PSD elements. Identifying characteristics associated with treatment efficacy will help optimize the design of future digital psychological interventions.

### Novel Contributions

Existing reviews of digital interventions in this area have not thoroughly considered the implementation and user experience of digital interventions, nor have they determined treatment effects for individual symptoms of BPD. Frias et al [31] conducted a scoping review of 15 studies of digital interventions in participants with BPD, which were primarily adjunctive to dialectical behavior therapy (DBT) but did not calculate their treatment effect. A meta-analysis of smartphone apps for BPD-related symptoms did not find a significant treatment effect. However, this meta-analysis pooled outcome measures across symptoms, potentially obscuring item-level changes for individual BPD symptoms [32]. Other reviews have focused on the user experience of these interventions, with a scoping review of 8 interventions, primarily DBT tools, reporting positive user feedback, while another review found serious issues with functionality and interface design, such as the exclusion of DBT diary cards, technical issues with enrollment, and difficulties with navigation [33,34]. This review provides a comprehensive evaluation of digital interventions that target both specific and transdiagnostic symptoms of BPD. Treatment effects for individual symptom measures and an analysis of the effect of user experience features and PSD elements on treatment efficacy are also examined.

## Methods

### Search Strategy

OVID Embase, OVID MEDLINE, OVID PsycINFO, and the Cochrane Central Register for Controlled Trials were searched for literature. The search strategy [35], further discussed in Multimedia Appendix 1, was developed in consultation with coauthor EH and consisted of three themes: (1) the core symptoms of BPD, (2) digital health, and (3) intervention studies. The digital health theme was based on a National Institute for Health and Care Excellence (NICE)–validated filter for health care applications, with additional terms added for web-based interventions [36]. Automatic filters were used to remove non-English language studies and those published before the year 2000, as the technology of interest did not exist before that time. The search was first executed on July 19, 2022, and was then re-executed on February 28, 2023, to check for new literature. Furthermore, we performed forward reference checking on the included publications to enhance the literature search.

### Eligibility Criteria

All records were screened by coauthors JABL and TH independently, and disagreements were settled in consultation with NMM. Rayyan (Rayyan Systems, Inc) was used to manage the screening process [37]. Studies were eligible if they were peer-reviewed, published in English after the year 2000, and assessed an automated digital intervention with a treatment target of BPD or one of its symptoms. Here, *automated* indicates an intervention for which content is primarily delivered without human support. Given the possibility of preliminary studies in this area, both randomized controlled trials (RCTs) and nonrandomized studies were eligible for inclusion in the descriptive analysis. All participants were aged ≥18 years. They were either healthy volunteers or individuals with a diagnosis of BPD or its common comorbidities of depressive disorders, anxiety disorders, substance abuse disorders, posttraumatic stress disorder, or complex posttraumatic stress disorder. Studies drawing on community samples were included because they tended to recruit participants with mild to moderate symptom severity, which corresponded with the degree of BPD symptom severity that would be reasonable to treat with an automated digital intervention. This aligns with the NICE guidelines for digital interventions, suggesting professional oversight should increase proportionally with clinical risk [38]. Exclusion criteria were as follows: (1) interventions without a primary digital component; (2) nonautomated interventions (eg, therapist-centered treatment via telehealth); (3) interventions requiring equipment inaccessible to the public (eg, functional magnetic resonance imaging or professional-caliber virtual or augmented reality); (4) participants recruited based on a mental health or neurodevelopmental disorder not mentioned in the inclusion criteria; or (5) studies focusing on participants belonging to special groups such as combat veterans, people who are displaced, or law enforcement officers. These groups were excluded because the life experiences of these participants were unlikely to be representative of a community sample of people with BPD.

### Quality Assessment

The risk of bias was assessed with Cochrane’s Risk of Bias 2 for RCTs including cluster-randomized trials and the National Institutes of Health Quality Assessment Tool for pre-post studies for all single-arm studies [39,40]. All studies were separately evaluated by coauthors JABL and TH, and discrepancies were settled by consensus.

### Extracted Data

The following features were extracted for each intervention: method of delivery, therapeutic approach, overall intended treatment duration and frequency of use, degree of human support, and any PSD elements present. Data were extracted from the published literature and, where possible, supplemented by direct access to publicly available interventions and interviews with authors. A reliability check of the coding of PSD elements was carried out in a random sample of 5 papers, resulting in 91% interrater reliability. Therapeutic approaches were coded individually to describe each study. To facilitate the subgroup analysis, therapeutic approaches were then classified into evidence-based treatments (EBTs) and non-EBTs. EBTs comprised DBT, cognitive behavioral therapy (CBT), mentalization-based treatment, acceptance and commitment therapy, schema therapy, and transference-focused therapy. The degree of human support of the intervention was binary coded for either full automation or some degree of support based on the descriptions provided in the publications. Facilitated interventions ranged from technical support to therapeutic support to adjunct to in-person care.

### PSD Elements

We used the coding scheme by Kelders et al [17] for PSD elements, based on a framework developed by Oinas-Kukkonen and Harjumaa [18] (Tables 1 and 2). All interventions were coded dichotomously, indicating the presence or absence of primary task, dialogue, and social support elements. In cases where the presence of PSD elements was unclear in the publication, we contacted the authors and accessed the intervention directly where possible. Failing these means, the element was coded as absent.

### Effect Sizes and Meta-Analysis

Treatment effects for the RCTs were calculated using between-group standardized mean differences (SMDs) at the study end point, with Hedges small sample correction applied [41]. Hedges *g* values of 0.0-0.2 were interpreted as small, 0.21-0.8 as moderate, and 0.81-1.00 as large. Outcome data were extracted directly from the publications or requested from the study lead author. In cases (2/32, 6%) where the authors did not reply or were unable to provide the requested data, the study was excluded from the meta-analysis [42,43].

Six separate meta-analyses were used to assess the effect of treatment for which there were at least 2 studies reporting these as outcomes. These involved the following outcomes: BPD symptom severity assessed via the Zanarini Rating Scale for Borderline Personality Disorder [44] and Borderline Personality Disorder Severity Index [45]; suicidal ideation via the Beck Scale for Suicidal Ideation (BSSI) [46], Suicidal Ideation Attributes Scale [47], Self-Injurious Thoughts and Behaviours Interview–Suicidal Ideation [48], Columbia-Suicide Severity Rating Scale [49], Suicidal Behaviours Questionnaire [50], and Suicide Status Form [51]; NSSI via the Self-Injurious Thoughts and Behaviours Interview–Nonsuicidal Self-Injury Episodes [48]; paranoia via the Paranoia Scale [52] and Adapted Paranoia Checklist [53]; and anger via the Modified Overt Aggression Scale [54] and Trait Anger Scale [55]. As emotion regulation is considered a central mechanism in BPD, a meta-analysis was also conducted with the following outcome measures: the Difficulties with Emotion Regulation Scale in both long and short forms [56] and the German version of the Emotion Regulation Skills Questionnaire [57]. Following the Cochrane Collaboration recommended procedures [58], in cases where multiple different outcome measures of the same symptom were used in a single study, the effect sizes of each measure were calculated and then aggregated before meta-analysis using the approach by Borenstein et al [59].

Random effects models were chosen over fixed-effects as considerable between-study heterogeneity was expected due to differences in control arms [60]. *I*^2^ was used to estimate between-study heterogeneity, and restricted maximum likelihood was used to estimate heterogeneity variance τ^2^ [61]. The Knapp-Hartung adjustment was applied, given the small number of studies in some of the meta-analyses [62]. Funnel plots were visually inspected for risk of publication bias. Egger tests were conducted in meta-analyses with sufficient studies [63].

### Subgroup Analysis

Following recommendations from Schwarzer et al [64] and Fu et al [65], in meta-analyses with ≥10 studies, subgroup analyses were conducted to determine the effects of specific intervention elements, provided there were a minimum of 4 studies per subgroup. Intervention features assessed using subgroup analysis included PSD elements, degree of human support, and use of EBT versus non-EBT interventions. τ^2^ was pooled across subgroups because of the small number of studies in some subgroups [59]. The Cochran *Q* test was used to evaluate the significance in subgroup differences with *P*<.10 considered significant for the *Q* test owing to low event rates [66-68]. The Benjamini-Hochberg false discovery rate was used to correct for multiple comparisons [69].

All meta-analytic procedures were done using the *R* (R Foundation for Statistical Computing) packages: *meta* (version 6.2.1)*, dmetar* (version 0.1.0), and *Mad* (version 0.8-3) packages [64,66,70].

### Registration

This systematic review was preregistered with PROSPERO (CRD42022358270). The PRISMA (Preferred Reporting Items for Systematic Reviews and Meta-Analyses) checklist can be found in Multimedia Appendix 2. Some deviations were made from the registered protocol: first, gray literature was not included. This decision was made after reviewing the gray literature, as we believed including it could lower the average quality of the studies and increase their overall risk of bias. Furthermore, most of the gray literature was not peer-reviewed and thus did not meet the inclusion criteria for this review. Adherence was not used as an outcome measure of the review because only 10% (4/42) of the included studies described adherence as the number of participants who completed the intervention. Other adherence metrics were reported, but these were inconsistent and could not be organized into a meaningful measure. Finally, some studies listed multiple outcome measures without identifying a primary measure. For this review, all studies reporting at least 1 appropriate outcome measure were included. Studies of interventions with multiple treatment targets and outcome measures were eligible to be included in multiple syntheses. For example, some interventions addressed both suicidal ideation and NSSI and thus are included in both syntheses.

## Results

### Search Results

**Figure 1:**
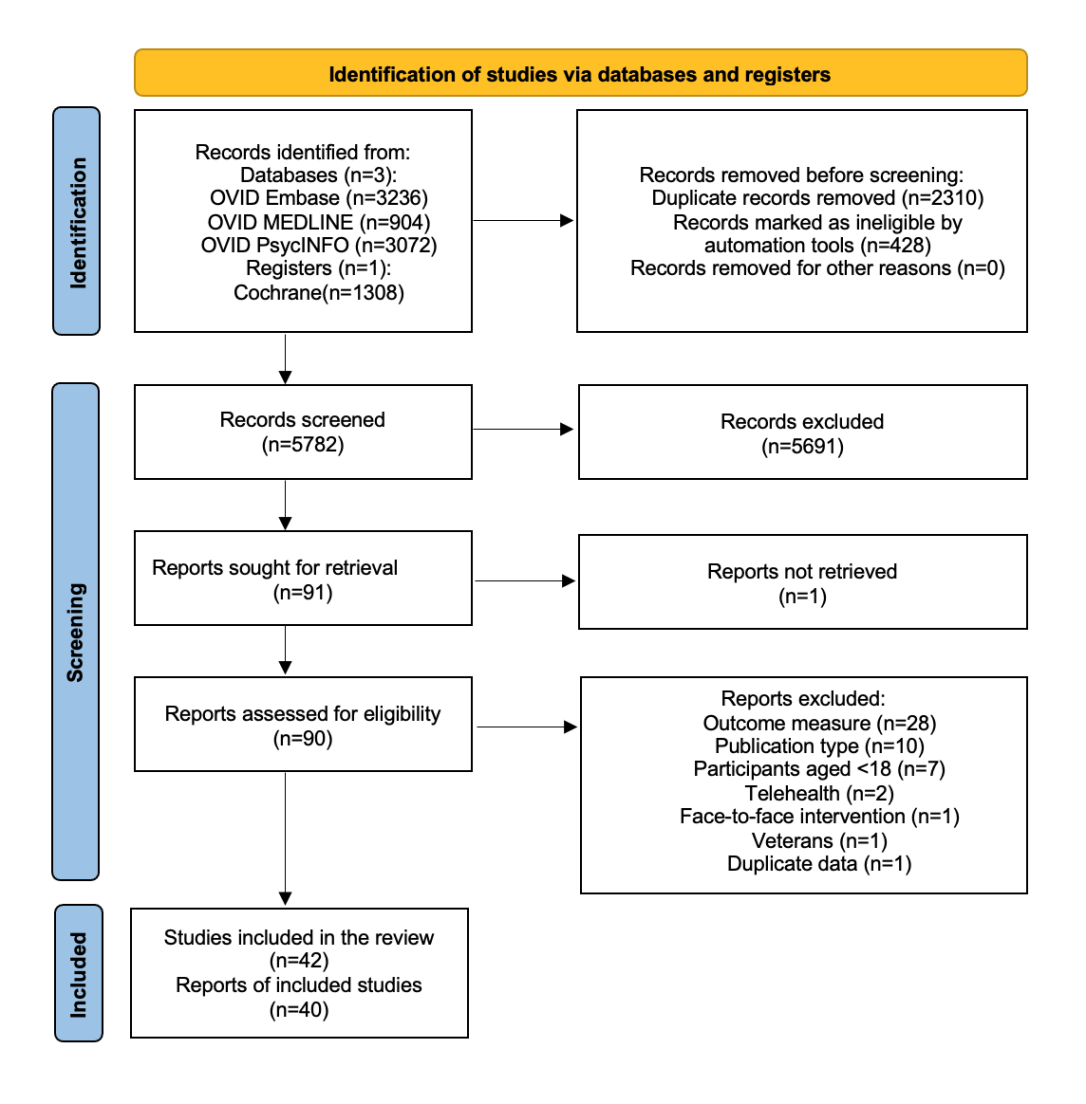
PRISMA (Preferred Reporting Items for Systematic Reviews and Meta-Analyses) flowchart showing the search and study selection process. For simplicity, the initial and repeat searches have been combined into 1 diagram.

Figure 1 illustrates the PRISMA flowchart of the initial and repeated searches. The first search, executed on July 19, 2022, returned 7531 records, of which 1645 (21.84%) were duplicates. A further 428 (5.68%) were automatically removed due to their year of publication (before 2000) or initial publication in a non-English language. Of the remaining 5458 records, 69 (1.26%) were retrieved for full-text screening and 36 (0.66%) of these were included. A repeated search before analysis with publication dates restricted from the initial search date to February 28, 2023, returned 989 records, of which 665 (67.24%) were duplicates. The remaining 324 records were screened, 22 (6.79%) were selected for full-text screening, and 4 (1.23%) of those were included. The details of all included studies are provided in Tables S1-S6 in Multimedia Appendix 1.

### Search Outcomes

The most common reason for exclusion was an inappropriate outcome measure, including unvalidated measures and subscales. Other reasons for exclusion were lack of peer review, enrollment of participants aged <18 years, interventions that were delivered in person or via telehealth meeting software, and research about combat veterans. A total of 40 reports met the eligibility requirements of this review, 1 (3%) of which detailed 3 studies in the same report, such that 42 unique studies were included. Across these studies, 38 unique digital interventions were assessed and discussed in this review. Some studies assessed the same intervention, for example, translations of Living with Deadly Thoughts into multiple languages [71-75]. One study had 3 arms, each assigned a different intervention [76]. In total, 38 unique interventions are discussed in this review. Of the 42 studies included in the review, 32 (76%) were RCTs, 8 (19%) were single-arm studies (pre-post), 1 (2%) was an open-label trial, and 1 (2%) was a cluster RCT. Four studies reported BPD symptoms or key mechanisms as outcome measures, 20 reported suicidal ideation and suicidal thinking, 5 studies each reported paranoia and NSSI, 4 reported emotion regulation, and 4 reported anger and hostility measures. Some interventions listed multiple outcome measures without indicating a primary. No interventions were identified targeting identity disturbance, impulsivity, feelings of emptiness, fears of abandonment, or relationship instability. Participants were recruited based on a variety of conditions: of the 6611 participants, 3372 (51%) were recruited based on suicidal ideation, 661 (10%) based on NSSI, 397 (6%) on a diagnosis of BPD, 331 (5%) on trait anger or hostility, and 198 (3%) on paranoia. In total, 19% (1256/6611) of the participants were recruited without specific baseline symptoms and 6% (396/6611) were recruited to studies with multiple possible symptom thresholds (eg, they met symptom thresholds for either suicidal ideation or NSSI).

### Risk of Bias

The results of the Cochrane’s Risk of Bias 2.0 and the National Institutes of Health Quality Assessment Tool for each study can be found in Figures S1-S5 in Multimedia Appendix 1. In brief, there was a widespread risk of bias: only 9% (3/32) of the RCTs and 13% (1/8) of the single-arm studies were deemed low risk.

### Intervention Features and System Design

The included studies were primarily conducted in the United States (16/42, 38%), the United Kingdom (6/42, 14%), or Australia (5/42, 12%). The methods of intervention delivery included websites (27/42, 64%), mobile phone apps (10/42, 24%), both (3/42, 7%), and email (2/42, 5%). The most common therapeutic approaches used were based on DBT (8/42, 19%), CBT (6/42, 14%), a combination of CBT and DBT (5/42, 12%), mindfulness (3/42, 7%), and acceptance and commitment therapy (2/42, 5%). Out of the 42 studies, 18 (43%) evaluated other or unspecified therapeutic approaches.

The mean course of treatment, unweighted by the number of participants, was 55 (SD 75) days, with a mean unweighted recommended frequency of use of 5 (SD 2.7) times per week. This frequency calculation excluded 26% (11/42) of the studies with open dosage or intended frequency of use not stated, as well as 10% (4/42) of single-session interventions.

Explanations for each PSD element and examples of relevant interventions are given in Tables 1 and 2, and the frequency of PSD elements used in each of the digital interventions is illustrated in Figure S6 in Multimedia Appendix 1. The presence of PSD elements varied from *suggestion,* used in 86% (36/42) of the studies to *praise*, for which we only found evidence in 10% (4/42) of the interventions. Elements of social support were not analyzed, as “e-motion” was the only intervention that allowed users to interact with one another [77].

### BPD Interventions

#### Study Characteristics

BPD psychopathology outcome measures were identified in 4 studies, including 3 RCTs, as outlined in Table S1 in Multimedia Appendix 1 [28,78-80]. Published between 2017 and 2021, these 4 studies recruited 376 participants in total, of which 303 (74%) provided follow-up data. Three of the studies had a moderate risk of bias [28,78,79], while 1 had a high risk of bias [80].

#### RCT Effect Sizes (Posttreatment SMDs, Hedges g)

**Figure 2:**
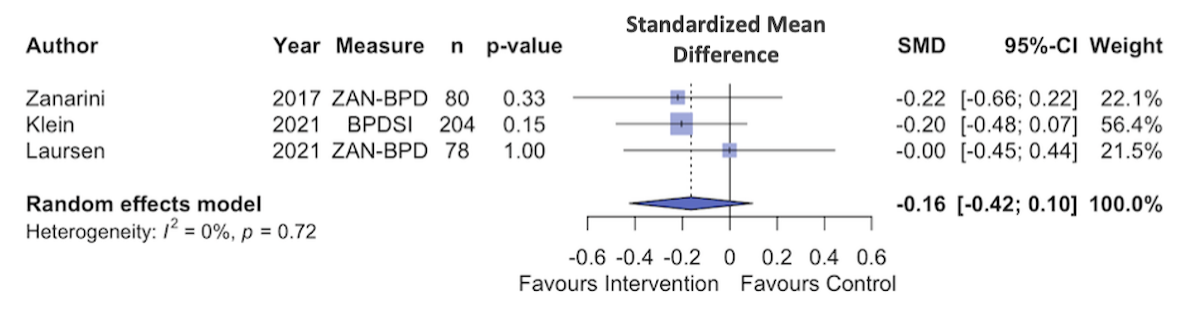
Forest plot of the treatment effect on borderline personality disorder symptom severity. SMD: standardized mean difference.

**Figure 3:**
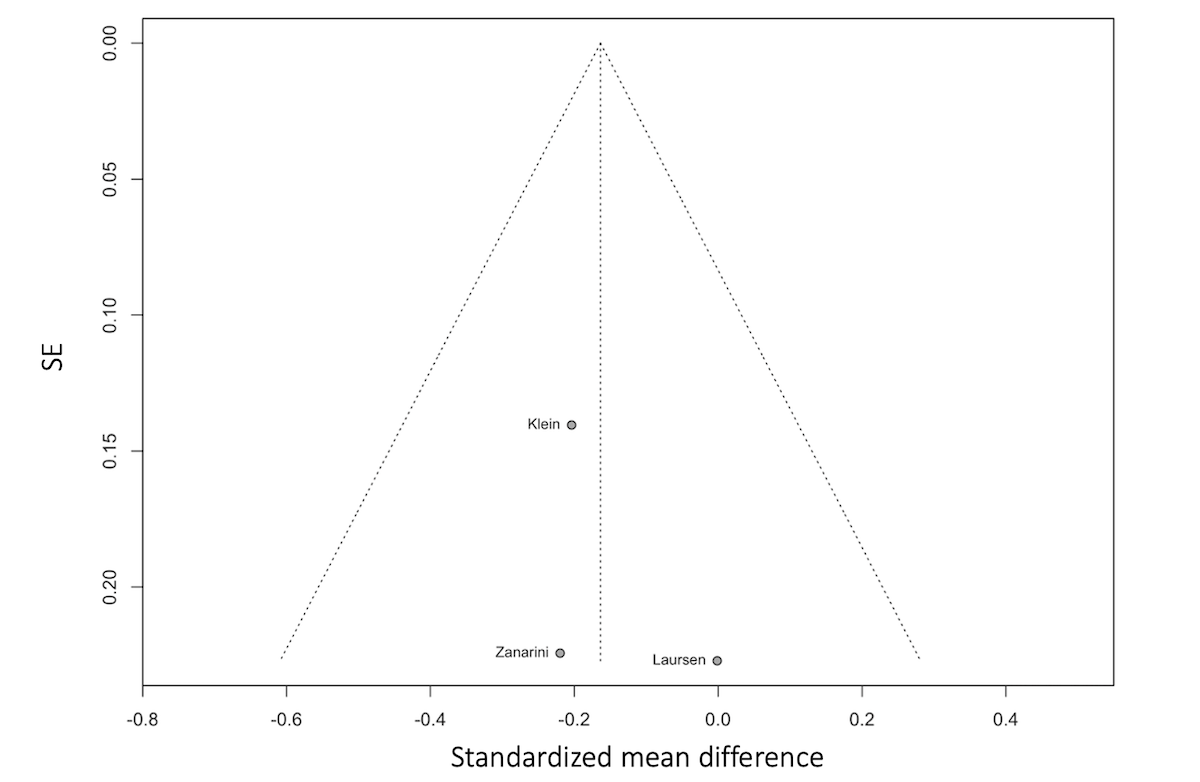
Funnel plot shows the treatment effect and SE of the interventions treating borderline personality disorder (BPD) psychopathology.

Data from the 3 RCTs was pooled, and a random effects model was fitted. Figure 2 [78-80] shows a small and nonsignificant treatment effect for digital interventions on BPD symptoms as a whole (N=3; Hedge *g*=–0.17, 95% CI –0.42 to 0.10; *P*=.11). The between-study heterogeneity variance was estimated at τ^2^=0 (95% CI 0.0-0.54) and *I*^2^=0% (95% CI 0%-89.6%). The funnel plot does not show evidence of publication bias (Figure 3 [78-80]).

### Suicidal Ideation Interventions

#### Study Characteristics

A total of 20 studies (18 RCTs) of interventions for suicidal ideation symptoms were identified, with publication years ranging from 2014 to 2023 and enrolling a total of 3983 participants (Table S2 in Multimedia Appendix 1 [21,22,25,27,30,71-76,80-86]). Of the 3983 participants, 2623 (66%) provided posttreatment data. Two (10%) of the studies were assessed at low risk of bias [75,81], 13 (65%) were at moderate risk of bias [21,25,27,71-74,76,82,83,86], and 5 (25%) were at high risk of bias [22,30,80,84,85].

#### Meta-Analysis

**Figure 4:**
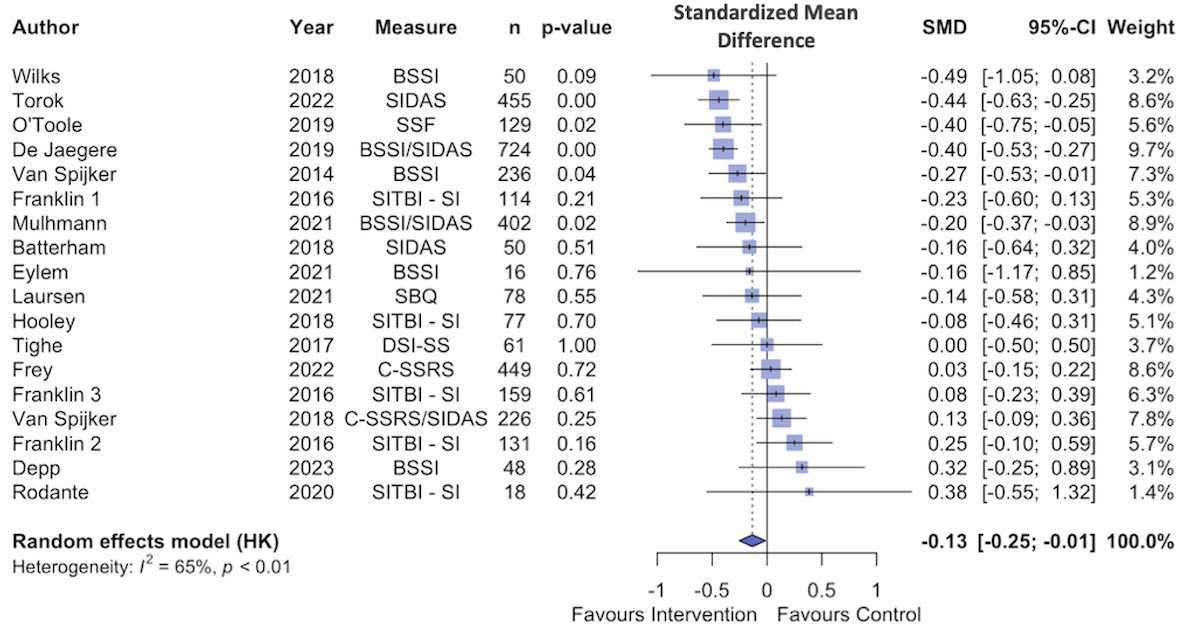
Forest plot of the treatment effect for suicidal ideation interventions. BSSI: Beck Scale for Suicidal Ideation; C-SSRS: Columbia-Suicide Severity Rating Scale; DSI-SS: Depressive Symptom Index–Suicidality Subscale; SBQ: Suicidal Behaviours Questionnaire; SIDAS: Suicidal Ideation Attributes Scale; SITBI-SI: Self-Injurious Thoughts and Behaviours Interview–Suicidal Ideation; SSF: Suicide Status Form.

**Figure 5:**
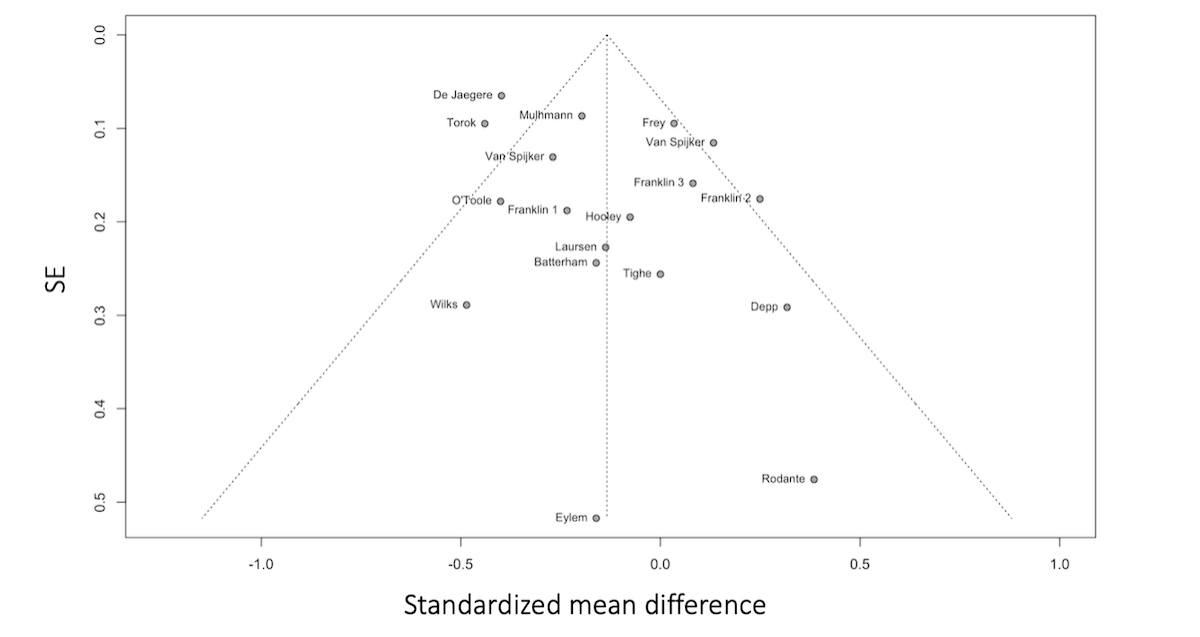
Funnel plot of the treatment effect and SE for suicidal ideation interventions.

As shown in Figure 4 [21,22,27,30,71-76,80-84,86], the random effects model fitted to the data found that digital interventions elicited a small, significant reduction in suicidal ideation (N=18; Hedges *g*=–0.13, 95% CI –0.25 to –0.01; *P*=.03). Between-study heterogeneity variance was estimated at τ^2^=0.3 (95% CI 0.01-0.10). Between-study heterogeneity was estimated at *I*^2^=64.7% (95% CI 41.6%-78.6%). As referenced in the Methods section, effect sizes in studies with multiple outcome measures of suicidal ideation were aggregated before the meta-analysis. The funnel plot in Figure 5 [21,22,27,30,71-76,80-84,86] does not show evidence of publication bias, and the Egger test also did not show evidence of bias (intercept=1.33, 95% CI –0.2 to 2.86; 2-tailed t_16_=1.70; *P*=.11).

### Subgroup Analysis: Effects of Suicidal Ideation Intervention Features

We first compared interventions targeting suicidal ideation that used EBTs (12/18, 67%) to those that did not (6/18, 33%). EBT interventions were associated with significantly greater treatment effect than non-EBT interventions (SMD –0.21 vs 0; Cochran Q=4.87; *P*=.03). We then compared suicidal ideation interventions that were fully automated to those that involved at least some degree of human support. There was not a significant difference in treatment effect between these 2 subgroups (automated SMD –0.09 vs facilitated SMD –0.16; Cochran Q=0.37; *P*=.54). Table 3 reports these results in detail, as well as subgroup analysis comparing treatment effect between interventions that did or did not use each PSD feature. For statistical power reasons, this analysis was only conducted for PSD elements that were present in at least 4 interventions and absent in at least 4 others. Interventions using reminders, opportunities for self-monitoring, and opportunities for rehearsal were associated with significantly greater reductions in suicidal ideation than those without. No significant effects were identified for other PSD elements.

**Table 3:** Difference in treatment effects between interventions using different therapeutic approaches, degrees of human support, and persuasive system design elements.

			Studies (n=18), n (%)		SMD^a^ (95% CI)		*P* value		*I*^*2*^ (%; 95% CI)		*P* value^b^	
**Therapeutic approach**												.03^c^
	EBT^d^	12 (67)		–0.21 (–0.37 to –0.06)		.01		61.4 (27.7-79.4)				
	Non-EBT	6 (33)		0.00 (–0.17 to 0.18)		.98		0.0 (0-74.6)				
**Support**												.54
	Human	12 (67)		–0.16 (–0.32 to –0.01)		.04		52.1 (7.67-75.2)				
	None	6 (33)		–0.09 (–0.35 to 0.17)		.42		80.1 (56.8-90.8)				
**Reduction**												.18
	Present	14 (78)		–0.18 (–0.32 to –0.04)		.02		65.3 (33.8-80.3)				
	Absent	4 (22)		0.01 (–0.31 to 0.34)		.90		23.5 (0-88.3)				
**Tunneling**												.29
	Present	5 (28)		–0.26 (–0.61 to 0.08)		.10		33 (0-74.6)				
	Absent	13 (72)		–0.10 (–0.24 to 0.04)		.13		68 (43.0-82.1)				
**Self-monitoring**												.095^c^
	Present	13 (72)		–0.20 (–0.35 to –0.06)		.01		56.1 (24.9-77.9)				
	Absent	5 (28)		0.01 (–0.21 to 0.24)		0.87		50.3 (0-80.4)				
**Rehearsal**												.08^c^
	Present	13 (72)		–0.21 (–0.36 to –0.07)		.01		58.2 (22.7-77.4)				
	Absent	5 (28)		0.02 (–0.19 to 0.23)		.80		0 (0-79.2)				
**Rewards**												.21
	Present	6 (33)		–0.01 (–0.29 to 0.28)		.95		37.4 (0-75.1)				
	Absent	12 (67)		–0.19 (–0.33 to –0.05)		.01		66.8 (39.1-81.9)				
**Reminders**												.08^c^
	Present	13 (72)		–0.21 (–0.35 to –0.07)		.01		58 (22.3-77.3)				
	Absent	5 (28)		0.03 (–0.17 to 0.24)		.67		0 (0-79.2)				
**Suggestion**												.18
	Present	14 (78)		–0.18 (–0.32 to –0.04)		.02		65.3 (38.8-80.3)				
	Absent	4 (22)		0.01 (–0.31 to 0.34)		.90		23.5 (0-88.3)				
**Liking**												.99
	Present	6 (33)		–0.13 (–0.39 to 0.12)		.23		65.6 (17.6-85.7)				
	Absent	12 (67)		–0.13 (–0.29 to 0.03)		.10		67.1 (39.6-82.0)				

^a^SMD: standardized mean difference.

^b^*P* value for Cochran *Q* (significant at *P*<.10) [58] comparing SMD between subgroups. *P* value has been corrected for multiple comparisons using the Benjamini-Hochberg method.

^c^Significance at *P*<.10.

^d^EBT: evidence-based treatment.

### Paranoia Interventions

#### Study Characteristics

From 42 studies, 5 (12%) RCTs of digital interventions were identified for paranoia, with publication years ranging from 2017 to 2021, totaling 413 enrolled participants (Table S3 in Multimedia Appendix 1 [87-91]). Many of the paranoia interventions were single sessions, so loss to follow-up percentage was not applicable. A total of 3 (60%) were at moderate risk of bias [87-89], while 2 (40%) were at high risk [90,91].

### Meta-Analysis

**Figure 6:**
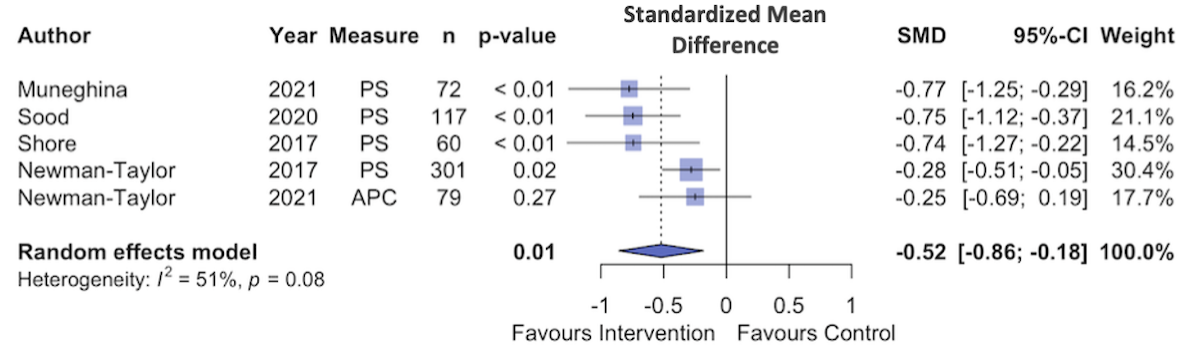
Forest plot showing standardized mean differences (SMDs) for interventions targeting paranoia. APC: Adapted Paranoia Checklist; PS: Paranoia Scale.

**Figure 7:**
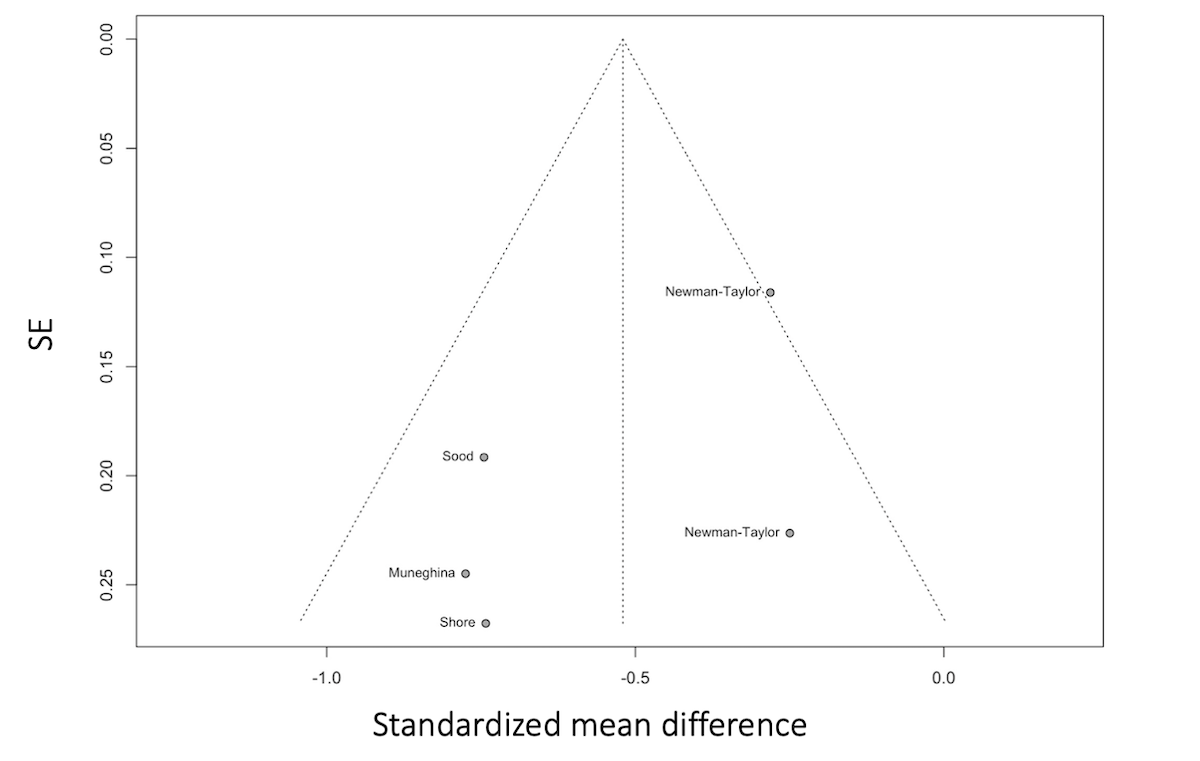
Funnel plot of the digital interventions targeting paranoia.

A random effects model fitted to the data found a moderate, significant effect of digital interventions for paranoia (N=4; Hedges g=–0.52, 95% CI –0.86 to –0.18; *P*=.01). The between-study heterogeneity variance was estimated at τ2=0.04 (95% CI 0-0.55), and between-study heterogeneity was estimated at I2=51.3% (95% CI 0%-82.1%; Figure 6 [87-91]). The funnel plot in Figure 7 [87-91] shows possible evidence of publication bias, but there were too few studies to conduct the test.

### Other BPD Symptoms

Meta-analysis of interventions targeting other symptoms (NSSI, emotion regulation, and anger) did not suggest a significant treatment effect of digital interventions. Full meta-analytic results for these interventions can be found in Figures S7-S12 in Multimedia Appendix 1. Identified interventions are tabulated in Multimedia Appendix 1: NSSI interventions are in Table S4 [27,43,76,86], emotion regulation interventions are in Table S5 [23,29,77,92-94], and anger interventions are in Table S6 [26,95,96].

## Discussion

### Principal Findings

This review suggests that digital interventions may be efficacious in reducing BPD symptoms of paranoia and suicidal ideation. By contrast, digital interventions addressing emotional regulation, anger, and NSSI did not demonstrate a significant treatment effect. Among interventions targeting suicidal ideation, those based on evidence-based therapeutic modalities for BPD were associated with greater improvement, but effectiveness was not found to be dependent on the degree of human support. Importantly, these findings highlight which symptoms may be better suited for digital interventions and could be candidates for further investigation and implementation. This is particularly relevant in clinically confirmed BPD, where limited access to in-person therapy and high service costs may be barriers to receiving timely care.

A secondary objective of this review was to assess the effect of PSD on treatment efficacy. There is an overlap between the aims of psychological interventions and the PSD framework: both support change in behavior and beliefs. At least some PSD element involvement was nearly ubiquitous among the studies reviewed; changes to thought and behavior (suggestion) and reduction of these changes into component processes (*reduction*) were used in 79% (33/42) and 86% (36/42) of the reviewed interventions, respectively. Importantly, our meta-analytic review of interventions targeting suicidal ideation found that the specific PSD elements *rehearsal*, *self-monitoring*, and *reminders* were associated with significantly greater treatment effect. Each element may confer different benefits to the design of digital interventions for suicidal ideation and per se parallel the therapeutic components of EBTs for suicidal ideation.

*Rehearsal* refers to practicing skills in a comfortable environment with the objective of building the habit of using them as needed. Rehearsing a response to periods of intense suicidal ideation may reduce the duration and risk of these events; this is a component in the Collaborative Assessment and Management of Suicidality approach [97]. Indeed, several evidence-based psychological interventions that address suicidal ideation such as DBT and CBT for suicide prevention involve rehearsal of applying the skills learned in therapy in advance of a challenging situation [98,99].

*Self-monitoring* in the context of PSD refers to keeping users motivated by encouraging reflection on progress toward goals. Both processes may address feelings of hopelessness and difficulty maintaining a course of action, which are associated with suicidal ideation and BPD [100,101]. Furthermore, self-monitoring, often with an emphasis on mood monitoring, is also a core aspect of interventions targeting emotional regulation and suicidality, such as DBT and CBT for suicide prevention.

*Reminders* are a design element that help reduce forgetful nonadherence [102]. Further work is necessary to understand which types of reminders are most effective for digital interventions. This PSD element is most likely to be digital-intervention specific, with no immediate comparative components in in-person EBTs.

In the context of existing therapeutic frameworks, these PSD features could be leveraged in the development of future digital interventions for suicidal ideation for BPD. Due care should be taken in the design of digital mental health interventions; the inclusion of PSD elements is a good starting point, but user experience should be evaluated and iterated upon, a process with established merits [103].

### Implications

As in face-to-face settings, digital EBT interventions for suicidal ideation, such as CBT and DBT, were more effective than alternative therapeutic approaches [104]. EBTs appear to be effective for reasons that transcend delivery method, perhaps by introducing active components that directly facilitate treatment goals, such as behavioral activation in CBT, rather than general nondirective support [105]. Despite the demonstrated efficacy of EBTs, some included interventions used promising alternative therapeutic approaches, such as stimulus pairing or journaling [27,76].

Efficacy of digital intervention may also vary depending on the treatment target. Included visualization interventions were effective, at least temporarily, in reducing symptoms of paranoia. Paranoia in BPD is transient, meaning immediate symptom reduction is relevant even if temporary. Recent work with virtual reality in community and psychosis samples has found that compassionate imagery, cognitive therapy, and mental relaxation exercises all appear to be effective in the reduction of paranoia symptoms [106,107]. The commonality between these approaches is reduced anxious cognitions, which likely underpin paranoia and form a suitable target for digital interventions [108,109]. All included paranoia interventions were brief (ranging from 1 session to 2 weeks). The NICE guidance for BPD advises against brief interventions for BPD partially because few brief EBTs were available at the time of the original 2009 publication, and partially because of the possibility of negative reaction upon withdrawal of therapist support [110]. The latter issue is less applicable to automated digital interventions, particularly if they remain accessible after treatment. Given the efficacy of paranoia interventions in this review, and as brief interventions minimize the risk of user attrition over time, we suggest that further research should evaluate brief digital interventions for symptom relief and teaching specific skills. Such interventions could be deployed in adjunct or anticipation of in-person treatment.

Suicidal ideation also appears feasible as a digital treatment target. For example, Living with Deadly Thoughts, an intervention based on the principles of CBT and DBT that has been translated into multiple languages, has been found effective in several large RCTs. Collectively, the studies of Living with Deadly Thoughts contributed 35% (1604/4583) of the meta-analytical suicidal ideation results. The intervention focuses on reducing negative automatic thoughts, which are associated with suicidal ideation through several mediation pathways [111]. Reduction in negative automatic thoughts after CBT is associated with reduced suicidal ideation [112], a treatment pathway that appears to have been successfully digitally adapted in this series of interventions. For interventions targeting paranoia and suicidal ideation, cognitive therapy components may be best suited to digital formats. These components can help reduce anxious thoughts in paranoia and negative automatic thoughts in suicidal ideation.

Unlike paranoia and suicidal ideation, meta-analytic evidence did not show improvement in emotion regulation, anger, or NSSI after digital intervention. DBT, the gold standard treatment for BPD, has been shown to improve these symptoms likely by improving behavioral control [9,10]. Behavioral control supports emotion regulation, including impulsive anger, which is common in BPD, and, in turn, predicts NSSI behaviors [9,11,113]. DBT typically involves protracted membership in a therapeutic group, through which patients learn to maintain relationships with peers. This process of supervised exposure and interaction with others is not replicable in automated digital interventions but may be essential to developing behavioral control. As a result, treatment targets of emotion regulation, anger, and NSSI may be less suited to the affordances of digital interventions than targets that can be effectively addressed through cognitive therapies, such as paranoia and suicidal ideation [12,114].

Previous research has found that human-supported digital mental health interventions are more effective than fully automated ones [14,115]. This was not replicated in our subgroup analysis of suicidal ideation interventions; however, human support could deter engagement with suicidal ideation treatments due to stigma and shame, thus reducing treatment efficacy for human-supported interventions [116]. This may not be true for other treatment targets; nonetheless, the effects of anonymity and human support should be weighed carefully in the development of digital interventions.

Our results suggest that effective digital interventions focus on building specific skills through transdiagnostic evidence-based approaches or provide symptom management tools [47]. This review did not find evidence that digital interventions should attempt comprehensive BPD treatment, as these interventions likely require support from a trained clinician and repeated opportunities to build emotion regulation skills among peers. The affordances of digital interventions are likely better suited to targeted, single-symptom treatments that could be deployed adjunctively to in-person care. For people with BPD, this type of intervention may be less pathologizing as the goal is to treat the symptoms of the individual rather than the disorder. The single-symptom approach is increasingly relevant as the diagnosis of personality disorder moves toward a dimensional model [117]. For users with and without personality disorders, the brevity and content relevance of transdiagnostic single-symptom interventions may improve adherence to digital interventions relative to extended courses of comprehensive treatment. Change in single-symptom severity may also be easier to measure because single-symptom measures provide more granularity than composite diagnostic measures [118].

### Limitations

There are several limitations to this review, the first being the limited applicability of findings across symptoms. For example, the association between PSD elements and treatment effect was only analyzed in interventions for suicidal ideation and may not hold for all treatment targets. In addition, while the scope of this review includes interventions for BPD symptoms, many of the included studies draw on non-BPD samples. As a result, our findings address the optimization of digital interventions for symptom reduction, rather than BPD psychopathology specifically. These results remain relevant to BPD given that treatment mechanisms in the disorder are poorly understood, and there is little evidence to suggest that efficacy in existing treatments is specific to BPD rather than a general reduction in psychiatric distress [119]. This work may also have been restricted by a lack of statistical power in some analyses, particularly the smaller meta-analyses of treatment effect and the subgroup analyses [66]. In addition, the binary coding of interventions into human-supported and fully automated subgroups may have obscured some of the effect of human support. For example, interventions that only offered as-needed support were grouped with interventions that included regular support, although the former might not impact the treatment effect as much as the latter. All negative subgroup results should be interpreted as an absence of evidence for efficacy rather than evidence of inefficacy. The meta-analyses and subgroup analyses also depend on the quality, completeness, and accuracy of the data in the included studies. As discussed, many studies were at risk of bias and had considerable loss to follow-up. Finally, when intervention descriptions were ambiguous or absent and authors did not reply to our queries, PSD elements were coded conservatively. This may have lowered the frequency estimates of PSD elements and the accuracy of the subgroup analyses.

### Conclusions

This review has found meta-analytic evidence that digital interventions, particularly cognitive interventions, are effective at reducing the BPD symptoms of paranoia and suicidal ideation. The most effective suicidal ideation interventions were those that used evidence-based therapies and certain features of PSD. Automated digital interventions are unlikely to replace face-to-face care as primary interventions for BPD because they do not facilitate sufficient opportunities to develop emotion regulation skills. However, digital interventions still represent an opportunity for highly accessible symptom-specific BPD treatment.

## Appendix

Multimedia Appendix 1 Details of the search strategy, studies included, nonsignificant meta-analyses of treatment effect, and subgroup analysis of population type (community vs outpatient).

Multimedia Appendix 2 PRISMA (Preferred Reporting Items for Systematic Reviews and Meta-Analyses) checklist.

## Abbreviations

BPD: borderline personality disorder
CBT: cognitive behavioral therapy
DBT: dialectical behavior therapy
EBT: evidence-based treatment
NICE: National Institute for Health and Care Excellence
NSSI: nonsuicidal self-injury
PRISMA: Preferred Reporting Items for Systematic Reviews and Meta-Analyses
PSD: persuasive system design
RCT: randomized controlled trial
SMD: standardized mean difference

## Data Availability

The datasets generated and analyzed during this study are available from the corresponding author on reasonable request.
