# Correction: Digital Interventions for Symptoms of Borderline Personality Disorder: Systematic Review and Meta-Analysis

**DOI:** 10.2196/70850

**Published:** 2025-02-28

**Authors:** Julia A B Lindsay, Niall M McGowan, Thomas Henning, Eli Harriss, Kate EA Saunders

**Affiliations:** 1 Department of Psychiatry University of Oxford Oxford United Kingdom; 2 School of Medicine and Biomedical Sciences University of Oxford Oxford United Kingdom; 3 Bodleian Health Care Libraries University of Oxford Oxford United Kingdom; 4 Oxford Health NHS Foundation Trust Warneford Hospital Oxford United Kingdom

In Digital Interventions for Symptoms of Borderline Personality Disorder: Systematic Review and Meta-Analysis J Med Internet Res 2024;26:e54941 there was an omission in published affiliations for one author and the Conflicts of Interest statement. We have also taken the opportunity to update the description of the medical school which has become part of the School of Medicine and Biomedical Sciences.

The affiliations for Saunders KEA were published as:

^1^Department of Psychiatry, University of Oxford, Oxford, United Kingdom

^2^John Radcliffe Medical School, University of Oxford, Oxford, United Kingdom

This has been amended to:

^1^Department of Psychiatry, University of Oxford, Oxford, United Kingdom

^2^School of Medicine and Biomedical Sciences, University of Oxford, Oxford, United Kingdom

^4^Oxford Health NHS Foundation Trust, Warneford Hospital, Oxford, United Kingdom

The affiliation for Henning T was published as:

^2^John Radcliffe Medical School, University of Oxford, Oxford, United Kingdom

This has been amended to:

^2^School of Medicine and Biomedical Sciences, University of Oxford, Oxford, United Kingdom

The conflicts of interest statement were published as:

JABL, KEAS, and NMM received nonfinancial support from Big Health Ltd in the form of no-cost access to the digital sleep improvement program, Sleepio, for use in clinical research, outside the submitted work. KEAS is supported by the National Institute for Health Research Oxford Biomedical Research Centre based at Oxford University Hospitals National Health Service Trust and the University of Oxford. NMM is a current employee of and holds shares, restricted share units, and/or share options in Compass Pathways plc; this work is not related to Compass Pathways plc.

This has been amended to:

JABL, KEAS, and NMM received nonfinancial support from Big Health Ltd in the form of no-cost access to the digital sleep improvement program, Sleepio, for use in clinical research, outside the submitted work. KEAS is supported by the National Institute of Health Research (NIHR) Oxford Health Biomedical Research Centre. NMM is a current employee of and holds shares, restricted share units, and/or share options in Compass Pathways plc; this work is not related to Compass Pathways plc. The views expressed are those of the authors and not necessarily those of the National Health Service, NIHR, or the Department of Health and Social Care.

The correction will appear in the online version of the paper on the JMIR Publications website on February 28, 2025, together with the publication of this correction notice. Because this was made after submission to PubMed, PubMed Central, and other full-text repositories, the corrected article has also been resubmitted to those repositories.

