# Supplementary material for: Digital Interventions for Symptoms of Borderline Personality Disorder: Systematic Review and Meta-Analysis
Source: J Med Internet Res. 2024 Nov 29;26:e54941. doi: 10.2196/54941 (PMC11645515; doi:10.2196/54941)
Supplement: Multimedia Appendix 1 [file jmir_v26i1e54941_app1.docx]

# Supplemental Material

Search Strategy

The search strategy, which was developed by co-authors JABL and EH, is reproduced below in full using Ovid Embase syntax, with the number of results returned at the end of each line. Terms related to BPD and its symptoms are captured by lines 1-12 and 30-44. Lines 13-24 were derived from a validated filter developed by the National Institute for Health and Care Excellence with the objective of capturing the many possible descriptors used for application-based digital interventions [36]. Lines 25, 26, and 45 were added to this filter to account for other delivery formats including web-based interventions, serious games, and interventions mislabeled as telehealth. Lines 27, 28, 50 and 52 capture terms related to symptoms and psychiatric illness. Lines 46-48 capture terms related to clinical trials and experimental settings. This final concept was added to the search to filter out observational studies. The search can be automatically executed by following the referenced link [35]. References were managed using EndNote 20 and Rayyan [37].

1 borderline state/ 15927

2 ((borderline or border-line) adj3 (personalit* or state*)).ti,ab,kw. 11882

3 ("Axis II" or "Cluster B" or flamboyant or "emotionally unstable personality" or EUPD or "F60.3" or "F60.30" or "F60.31").kw,tw. 4678

4 (idealization adj5 devaluation).kw,tw. 34

5 ((vulnerable or hyberbolic) adj3 temperament).kw,tw. 12

6 (((unstab* or instab* or poor or disturb* or fail* or weak or dysregulat*) adj3 (self* or impuls* or interperson* or identit* or relationship* or emotion* or affect*)) and (personality or character or PD)).kw,tw. 4918

7 (impulsiv* adj5 (behavio?r or character or personalit*)).kw,tw. 6309

8 (self adj3 (injur* or damag* or destruct* or harm* or hurt* or mutilat*)).kw,tw. 27728

9 (suicidal adj3 (behavior or behaviour)).kw,tw. 13793

10 (feel* adj3 (empt* or bored*)).kw,tw. 607

11 (anger adj5 control*).kw,tw. 1465

12 (risk-taking adj3 (behavior or behaviour)).kw,tw. 2785

13 exp mobile application/ 25719

14 exp mobile phone/ 47811

15 text messaging/ 7699

16 personal digital assistant/ 1833

17 computer assisted therapy/ 4861

18 (app or apps).ti,ab. 59462

19 ((online or web or internet or digital* or virtual) adj3 (based or application* or intervention* or program* or therap*)).ab. 119319

20 (phone* or telephone* or smartphone* or cellphone* or smartwatch*).ti. 32393

21 ((phone* or telephone* or smartphone* or cellphone* or smartwatch*) adj3 (based or application* or intervention* or program* or therap*)).ab. 22835

22 (mobile health or mhealth or m-health or ehealth or e-health or emental or e-mental).ti. 9450

23 ((mobile health or mhealth or m-health or ehealth or e-health or emental or e-mental) adj3 (based or application* or intervention* or program* or therap*)).ab. 6510

24 (mobile* adj3 (based or application* or intervention* or device* or technolog*)).ti,ab. 27130

25 exp telemedicine/ 71490

26 (telemedicine or tele-medicine or telehealth or tele-health or wearable*).ti,ab. 66756

27 symptom*.ti,ab,kw. 2128090

28 exp symptom/ 169701

29 27 or 28 2173329

30 borderline state/ 15927

31 "borderline psychosis".tw,kw. 16

32 abandon*.tw,kw. 34055

33 ("interpersonal effectiveness" or "interpersonal sensitivity" or "interpersonal hypersensitivity" or "rejection sensitivity" or "rejection hyper-sensitivity").tw,kw. 2145

34 ("identity disturbance" or "uncertain sense of self" or "shifting identity").tw,kw. 184

35 impulsiveness/ 27235

36 impulsiv*.tw,kw. 34929

37 exp suicidal behavior/ 127019

38 exp automutilation/ 24507

39 (self-injur* or selfinjur* or "self harm*" or "self-inflicted injur*" or selfinflict* or para-suicid* or parasuicid* or suicid*).tw,kw. 133863

40 ("emotion dysregulat*" or "emotional dysregulat*" or "emotion regulat*" or "emotional regulat*" or "affective instabil*" or "affective labil*" or "mood instabil*" or "mood labil*" or "aversive tension").kw,tw. 22796

41 anger management therapy/ or anger/ 22458

42 (anger or angry).tw,kw. 29497

43 paranoia/ 11981

44 (depersonaliz* or depersonalis* or derealiz* or derealis* or dissociat* or paranoi* or suspicio*).tw,kw. 368290

45 ("serious game*" or gamifi* or "applied game*").tw,kw. 3106

46 exp clinical trial/ or "clinical trial (topic)"/ or clinical trial protocol/ 1954147

47 human experiment/ or therapeutic research/ 642337

48 (treatment* or task* or therap* or pre-therap* or post-therap* or interven* or train* or tool*).kw,tw. 12945540

49 46 or 47 or 48 13970931

50 (psychopathology or risk* or prevention or crisis or crises or psychiatr* or disorder* or "psychological distress" or "psychological state" or "mental health" or harm*).kw,tw. 6934344

51 1 or 2 or 3 or 4 or 5 or 6 or 7 or 8 or 9 or 10 or 11 or 12 or 30 or 31 or 32 or 33 or 34 or 35 or 36 or 37 or 38 or 39 or 40 or 41 or 42 or 43 or 44 686992

52 symptom/ 164738

53 mental disease/ 277629

54 27 or 28 or 50 or 52 8263048

55 13 or 14 or 15 or 16 or 17 or 18 or 19 or 20 or 21 or 22 or 23 or 24 or 25 or 26 or 45 357217

56 49 and 51 and 54 and 55 3524

57 limit 56 to english language 3455

58 limit 57 to yr="2000 -Current" 3429

## Study Characteristics, presented by symptom

### Tabulation of BPD-Specific Interventions

Four interventions were identified which listed their treatment targets as BPD broadly rather than a specific symptom (Table S1). These studies, published from 2017 to 2021, include data from 376 participants.

Table S1: Study Characteristics of BPD Interventions

| **Author, Year, Location, Type of study** | ***Intervention name*, therapeutic approach, duration, frequency & access method.**  **Degree of Human Facilitation.** | **Population, Enrolment/Assignment, Loss to follow-up, Comparator** | **Outcome measure(s) & Summary** | **PSD Elements Employed** |
| --- | --- | --- | --- | --- |
| Jacob, Hauer [28] 2018. Germany,  Single arm pre-post pilot study.  2018. | *priovi,* schema therapy, web.  Adjunctive to in-person treatment. | Outpatients with BPD diagnosis  14 enrolled, 2 (14%) lost to FU | Significant reductions in BPDSI & BPD-CL scores | Reduction  Tunneling  Tailoring  Personalisation  Reminders  Suggestion  Similarity  Liking |
| Klein, Hauer-von Mauschwitz [79] 2021. Germany,  RCT. | *priovi,* schema therapy, 1 year, twice weekly, web.  Technical support only. | Outpatients with diagnosis or probable diagnosis of BPD  Digital intervention arm*:* 103 assigned, 42 (41%) lost to FU  Control (TAU) arm: 101 assigned, 26 (26%) lost to FU | BPD symptom frequency (BPDSI) had significantly greater decrease in *priovi* group | Reduction  Tunneling  Tailoring  Personalisation  Reminders  Suggestion  Similarity  Liking  Rewards |
| Laursen, Helweg-Jørgensen [80] 2021. Denmark,  RCT & economic evaluation | *mDiary*, DBT, 40 weeks to 1 year depending on study site, daily, mobile application.  Adjunctive to in-person treatment. | Outpatients with EUPD (F60.3)  Digital intervention arm*:*  42 assigned  Active control arm: 36 assigned  Drop-out acknowledged but not reported. | Between-group differences in ZAN-BPD scores were insignificant | Reduction  Self-monitoring  Rehearsal  Reminders  Suggestion  Liking |
| Zanarini et al. [78] 2017. USA,  RCT | (none), DBT, 6 weeks, weekly, web.  None. | Community sample of women meeting diagnostic criteria for BPD  Digital intervention arm*:* 40 assigned, 1 (2.5%) lost to FU.  Control arm: 40 assigned, 2 (5%) lost to FU. | ZAN-BPD: no significant difference in total score between groups.  BEST: no significant difference between groups | Interface not described in text and no response from authors |

### Tabulation of Suicidal Ideation Interventions

Twenty studies targeting symptoms of suicidal ideation were identified (Table S2), with publication years ranging from 2016 to 2023. These studies include data from 3,983 participants.

Table S2: Study characteristics of suicidal ideation interventions

| **Author, Year, Location,**  **Type of study** | ***Intervention name*, therapeutic approach, duration, frequency, access method,**  **human facilitation** | **Population, Enrolment, Attrition & Adherence** | **Outcome measure(s) & Summary** | **PSD Elements Employed** |
| --- | --- | --- | --- | --- |
| Batterham, Calear [30]. 2018. Australia,  RCT (3 arms) | *FitMindKit*, multiple,  2 weeks,  daily,  website access.  None. | Community sample of adults with moderate mood disorder symptoms.  Digital intervention static version: 62 randomised, 41 (66%) lost to follow-up.  Digital intervention tailored version: 66 randomised, 41 (62%) lost to follow-up.  Control arm: 66 randomised, 37 (56%) lost to follow-up. | SIDAS: no significant decreases or time x condition effects in suicidal ideation. | Reduction  Tunneling  Rehearsal  Reminders  Suggestion  Similarity  Liking  Social Role |
| Crosby and Witte [25] 2021. USA,  Single arm | *Sleep Scholar,* CBT-I,  1 session + 14 sleep diaries, website access,  None. | University students with insomnia & suicidal ideation  40 enrolled, 2 (5%) excluded due to technical difficulties, 5 (13%) lost to follow-up. | DSI-SS: No significant reduction in suicidal ideation | Reduction  Tailoring  Personalisation  Self-monitoring  Simulation  Rewards  Suggestion |
| De Jaegere, van Landschoot [72] 2019. Belgium,  RCT | *Think Life* (adaptation of Living with Deadly Thoughts)*,*  CBT,  6 weeks,  weekly,  website access,  None. | Adults with suicidal ideation.  Digital intervention arm*:* 365 randomised, 270 (74%) lost to follow-up.  Control arm: 359 randomised, 187 (52%) lost to follow-up. | BSS & SIDAS: Significantly greater reduction in both measures of suicidal ideation for the *Think Life* group. | Reduction  Self-monitoring  Rehearsal  Reminders  Suggestion |
| Depp, Parrish [83] 2023.  USA,  RCT (pilot) | *mSTART,*  CBT,  3 months,  daily use,  mobile application, adjunctive to in-person treatment. | Outpatients with SMIs & suicidal ideation  Digital intervention arm*:* 38 assigned, 11 (29%) lost to follow-up.  In-person only: 40 assigned, 10 (25%) lost to follow-up. | BSSI: No significant effect of group at post-treatment | Reduction  Tailoring  Personalisation  Self-monitoring  Simulation  Rehearsal  Rewards  Suggestion |
| Eylem, van Straten [71] 2021.  Netherlands & UK,  RCT | *Living with Deadly Thoughts (Turkish version),*  6 weeks,  weekly,  website access,  regular content support. | Community sample of adults with suicidal ideation.  Digital intervention arm*:* 10 randomised, 0 lost to follow-up.  Control arm: 8 randomised, 2 (25%) lost to follow-up. | BSS:  No significant time x condition effects. | Reduction  Self-monitoring  Rehearsal  Reminders  Suggestion |
| Frey, Osteen [22] 2023.  USA,  RCT | *Man Therapy,* stigma reduction, 3 months open dosage,  website,  none. | Community adults with suicidal ideation.  Digital intervention arm*:* 279 assigned, 72 (26%) lost to follow-up.  Sham control: 275 assigned, 33 (12%) lost to follow-up. | C-SSRS: no significant effect of group at post-treatment. | Reduction  Tailoring  Personalisation  Simulation  Suggestion  Similarity  Liking  Social Role |
| Franklin, Fox [27] 2016. USA,  RCT | *TEC (Study 1 version)*, evaluative conditioning,  2 months,  open dosage, mobile application,  none. | Adults with recent self-cutting    Active digital intervention arm*:* 55 randomised, 14 (25%) lost to one-month follow-up.    Sham digital intervention arm: 59 randomised, 21 (36%) lost to one-month follow-up. | Significantly fewer general NSSI episodes, self-cutting episodes, and suicidal plans in the active group than the sham group. | Rewards |
| Franklin, Fox [27] 2016. USA,  RCT | *TEC (Study 2 version)*, evaluative conditioning,  2 months,  open dosage, mobile application,  none. | Adults with recent self-cutting    Active digital intervention arm*:* 62 randomised, 18 (29%) lost to follow-up.    Sham digital intervention arm*:* 69 randomised, 17 (25%) lost to follow-up. | Significantly fewer self-cutting episodes in the active group as compared to the sham group. No difference in self-cutting events (ie. Total number of cuts) or general NSSI episodes. | Rewards |
| Franklin, Fox [27] 2016. USA,  RCT | *TEC (Study 3 version)*, evaluative conditioning,  2 months,  open dosage, mobile application,  none. | Community sample of adults with suicidal ideation.  Active digital intervention arm*:* 75 randomised, 39 (52%) lost to one-month follow-up.  Control digital intervention arm*:* 84 randomised, 30 (36%) lost to one-month follow-up. | SITBI:  Significant reduction in suicide plans for active group, no significant difference in ideation between groups | Rewards |
| Hooley, Fox [76] 2018.  USA,  RCT (3 arms) | *Autobiographical Self-Enhancement Training (ASET), Expressive Writing (EW),*  journaling,  1 month,  daily,  website access,  as-needed treatment support. | Adults with recent NSSI    ASET: 49 randomised, 8 (16%) lost to one-month follow-up.    EW: 49 randomised, 4 (8%) lost to one-month follow-up.    Basic journaling control: 46 randomised, 9 (20%) lost to one-month follow-up. | SITBI – NSSI & suicidal ideation frequency: Basic journaling group had significantly fewer days of suicidal ideation than EW group. No other significant effects. | Reduction  Self-monitoring  Rehearsal  Reminders  Suggestion |
| Laursen, Helweg-Jørgensen [80] 2021. Denmark,  RCT & economic evaluation | *mDiary*,  DBT,  40-52 weeks depending on study site,  daily,  mobile application,  adjunctive to in-person treatment. | Outpatients with EUPD (F60.3)  Digital intervention arm*:* 42 assigned  Paper diary control arm: 36 assigned  Drop-out acknowledged but not reported. | Unadjusted between-group differences in suicidal behaviour (SBQ) were insignificant | Reduction  Self-monitoring  Rehearsal  Reminders  Suggestion  Liking |
| Mühlmann, Madsen [73] 2021. Denmark,  RCT | *Living with Deadly Thoughts (Danish version)*,  CBT,  6 weeks,  daily,  website access,  as-needed content support | Community sample of adults with suicidal ideation.  Digital intervention arm*:* 196 randomised, 15 (8%) lost to follow-up.  Control arm: 206 randomised, 24 (12%) lost to follow-up. | BSS & SIDAS: Significant reduction in both measures of suicidal ideation for intervention arm | Reduction  Self-monitoring  Rehearsal  Reminders  Suggestion |
| O'Toole, Arendt [84] 2019. Denmark,  RCT | *Life App’tite*,  8 weeks,  daily,  mobile application,  adjunctive to in-person treatment. | Outpatients with suicidal ideation.  Digital intervention arm*:* 60 randomised, 34 (57%) lost to follow-up.  Control (TAU) arm: 69 randomised, 30 (43%) lost to follow-up. | SSF: Significant time x condition effect on suicide risk. | Reduction  Self-monitoring  Rehearsal  Suggestion |
| Pauwels, Aerts [85] 2017. Belgium,  Single arm pre-post | *BackUp*, crisis planning, 1 week, open dosage, mobile application,  none. | Community sample of adults with suicidal ideation.  45 enrolled, 24 (53%) dropped out. | BSS, non-significant decrease in suicidal ideation | Reduction  Personalisation  Suggestion  Liking |
| Rodante, Kaplan [86] 2022. Argentina,  Cluster RCT | *Calma,*  DBT,  1 month,  open dosage, mobile application,  adjunctive to in-person treatment | Outpatients with suicidal ideation  Digital intervention + DBT: 11 randomised & allocated, 2 (18%) lost to follow-up.  DBT only: 11 randomised & allocated, 1 (9%) lost to follow-up. | No significant differences between groups. | Reduction  Tunneling  Tailoring  Self-monitoring  Rehearsal  Reminders  Suggestion  Liking |
| Tighe, Shand [81] 2017. Australia,  RCT | *iBobbly*,  ACT,  6 weeks,  open dosage, mobile application,  none. | Young indigenous adults with suicidal ideation and depression/distress.  Digital intervention arm*:* 31 randomised, 2 (6%) lost to follow-up.  Control arm: 30 randomised, 0 lost to follow-up. | DSI-SS: significant decrease in suicidal ideation for intervention arm, but no time x condition effect. | Reduction  Tunneling  Personalisation  Self-monitoring  Rehearsal  Suggestion  Similarity  Liking |
| Torok, Han [21] 2022. Australia,  RCT | *Life Buoy*,  DBT,  6 weeks,  open dosage, mobile application,  none. | Community adults with suicidal ideation  Digital intervention arm*:* 228 randomised, 66 (29%) lost to follow-up.  Sham control arm: 227 randomised, 57 (25%) lost to follow-up. | SIDAS: Significant reduction in suicidal ideation scores & significant time x condition effect in intervention arm. | Reduction  Tunneling  Self-monitoring  Simulation  Rehearsal  Reminders  Suggestion  Liking |
| van Spijker, van Straten [74] 2018. Netherlands,  RCT | *Living with Deadly Thoughts (Dutch version)*,  CBT & DBT,  6 weeks,  daily,  website access,  none. | Adults with mild to moderate suicidal ideation.  Digital intervention arm*:* 116 randomised, 11 (9%) lost to follow-up  Control arm: 120 randomised, 10 (8%) lost to follow-up. | BSS: Significant reduction in suicidal in intervention arm | Reduction  Self-monitoring  Rehearsal  Reminders  Suggestion |
| van Spijker, Werner-Seidler [75] 2018.  Australia,  RCT | *Living with Deadly Thoughts (English version)*,  CBT & DBT,  6 weeks,  daily,  website access,  crisis support as needed. | Community adults with current suicidal ideation of any severity.  Digital intervention arm*:* 207 randomised, 90 (43%) lost to follow-up.  Sham control arm: 211 randomised, 102 (48%) lost to follow-up. | No group differences in suicidal ideation. | Reduction  Self-monitoring  Rehearsal  Reminders  Suggestion |
| Wilks, Lungu [82] 2018.  USA,  RCT | *None,*  iDBT-ST,  8 weeks,  weekly,  website access,  technical support as-needed. | University students with suicidal ideation, heavy drinking & emotion dysregulation.  Digital intervention arm: 30 enrolled  Control arm: 29 enrolled  In total, 21 (36%) were lost to follow-up. | SSI & DERS: No significant time x condition effects on suicidal ideation or emotion regulation. | Reduction  Tunneling  Self-monitoring  Rehearsal  Rewards  Suggestion |

### Tabulation of Paranoia Interventions

Five studies of interventions targeting paranoia were identified, with publication years ranging from 2018 to 2021 and enrolling a total of 679 participants (Table S3).

Table S3: Study characteristics for interventions targeting paranoia

| **Author, Year, Location, Study Type.** | ***Intervention name*, modality, duration, frequency, access method,**  **human facilitation** | **Population, Enrolment, Attrition & Adherence** | **Outcome measure(s) & Summary** | **PSD Elements Employed** |
| --- | --- | --- | --- | --- |
| Muneghina, Van Gordon [87] 2021,  UK,  RCT. | *Nature Based Intervention (NBI),* mindfulness,  5 days,  daily,  website access,  none. | Community sample of healthy adults  Digital intervention arm: 37 allocated, 0 lost to follow-up  Control arm: 35 allocated, 0 lost to follow-up | Significant time x condition effect on Paranoia Scale scores | Rehearsal  Suggestion  Liking |
| Sood and Newman-Taylor [88], 2020,  UK,  RCT. | *No name,* attachment-based imagery, 1 session, website access,  none. | Community & university sample with elevated but non-clinical paranoia  Digital intervention arm: 61 allocated  Active control arm: 56 allocated | Significant time x condition effect on Paranoia Scale score | Suggestion |
| Shore, Strauss [90], 2018,  UK ,  RCT. | *Learning Meditation Online,* mindfulness,  2 weeks,  open dosage, website access,  as-needed technical support. | Community & university sample of healthy adults  Digital intervention arm: 56 randomised, 27 (48%) lost to follow-up. Mean days of use: 11.83 (SD = 3.68)  Active control arm: 54 randomised, 25 (46%) lost to follow-up | Significant time x condition effect on Paranoia Scale score | Reduction  Rehearsal  Suggestion |
| Newman-Taylor, Kemp [91], 2018,  UK,  RCT. | *No name,* attachment-based imagery, 1 session, website access,  none. | University sample of healthy adults  Digital intervention arm: 140 allocated  Active control arm: 161 allocated | No significant effect on Paranoia Scale scores | Suggestion |
| Newman-Taylor, Sood [89], 2021,  UK,  RCT. | *None,* attachment-based imagery, 5 days,  daily,  website access,  none. | Community & university sample with elevated but non-clinical paranoia  Digital intervention arm: 42 allocated, 0 lost to follow-up  Active control arm: 37 allocated, 0 lost to follow-up | Significant time x condition effect on Paranoia Scale score | Suggestion |

### Tabulation of Non-Suicidal Self-Injury

Six studies of interventions for NSSI were identified, with publication years ranging from 2016 to 2020 and enrolling a total of 679 participants (Table S4). All studies were assessed at moderate risk of bias (19, 30-32).

Table S4: Study characteristics of NSSI Interventions

| **Author, Year, Location, Type of Study** | ***Intervention name*, modality, duration, frequency & access method.**  **Degree of Human Facilitation.** | **Population, Enrollment, Attrition & Adherence** | **Outcome measure(s) & Summary** | **PSD Elements Employed** |
| --- | --- | --- | --- | --- |
| Franklin et al. [27] 2016, USA,  RCT | *TEC (Study 1 version)*, evaluative conditioning, 2 months, open dosage, mobile application.  None. | Adults with recent self-cutting    Active *TEC* arm: 55 randomised, 14 (25%) lost to one-month follow-up.    Sham *TEC* arm: 59 randomised, 21 (36%) lost to one-month follow-up. | Significantly fewer general NSSI episodes, self-cutting episodes, and suicide plans in the active group than the sham group. | Rewards |
| Franklin et al. [27] 2016, USA,  RCT | *TEC (Study 2 version)*, evaluative conditioning, 2 months, open dosage, mobile application.  None. | Adults with recent self-cutting    Active *TEC* arm: 62 randomised, 18 (29%) lost to follow-up.    Sham *TEC* arm: 69 randomised, 17 (25%) lost to follow-up. | Significantly fewer self-cutting episodes in the active group as compared to the sham group. No difference in self-cutting events (ie. Total number of cuts) or general NSSI episodes. | Rewards |
| Franklin, Fox [27] 2016, USA,  RCT | *TEC (Study 3 version)*, evaluative conditioning,  2 months,  open dosage, mobile application,  none. | Community sample of adults with suicidal ideation.  Active digital intervention arm*:* 75 randomised, 39 (52%) lost to one-month follow-up.  Control digital intervention arm*:* 84 randomised, 30 (36%) lost to one-month follow-up. | SITBI:  Significant reduction in suicide plans for active group, no significant difference in ideation between groups | Rewards |
| Hooley et al. [76] 2018, USA,  RCT | *Autobiographical Self-Enhancement Training (ASET), Expressive Writing (EW),* journaling, 1 month, daily, website access.  Treatment support, as-needed. | Adults with recent NSSI    ASET: 49 randomised, 8 (16%) lost to one-month follow-up.    EW: 49 randomised, 4 (8%) lost to one-month follow-up.    Basic journaling control: 46 randomised, 9 (20%) lost to one-month follow-up. | SITBI – NSSI & suicidal ideation frequency: Basic journaling group had significantly fewer days of suicidal ideation than EW group. No other significant effects. | Self-monitoring,  Rewards,  Reminders |
| Drabu et al. [43] 2022, Singapore,  RCT | None, self-compassion training, 1 week, daily, audio recordings accessible online.  None | Adults with NSSI and self-criticism    Intervention arm: 30 randomised, 0 discontinued    Control arm: 33 randomised, 0 discontinued. | SITBI: Significant time x condition effect on inclination to self-injure | Self-monitoring,  Rehearsal,  Suggestion |
| Rodante [86] 2020, Argentina,  Cluster RCT | *Calma,*  DBT,  1 month,  open dosage, mobile application,  adjunctive to in-person treatment | Outpatients with suicidal ideation  Digital intervention + DBT: 11 randomised & allocated, 2 (18%) lost to follow-up.  DBT only: 11 randomised & allocated, 1 (9%) lost to follow-up. | No significant differences between groups. | Reduction  Tunneling  Tailoring  Self-monitoring  Rehearsal  Reminders  Suggestion  Liking |

### Tabulation of Emotion Regulation Interventions

Six studies (4 RCTs) of interventions for emotion regulation were identified, with publication dates ranging from 2011-2021 and enrolling a total of 599 participants (Table S5). Of these, one study was assessed to have low risk of bias (33), three at moderate risk of bias (34-36) and two at high risk of bias (37, 38).

Table S5: Study Characteristics of emotion regulation interventions

| **Author, Year, Location,**  **Type of study** | ***Intervention name*, modality, duration, frequency & access method.**  **Degree of Human Involvement in Treatment.** | **Population, Enrollment, Attrition & Adherence** | **Outcome measure(s) & Summary** | **PSD Elements Employed** |
| --- | --- | --- | --- | --- |
| Flujas-Contreras et al. [92] 2021, Spain,  Single-arm pre-post | *Parenting Forest,* ACT, 6 weeks, weekly, website access.  Reminders only. | Parents, either with inflexible parenting or with children with behavioural difficulties.  27 enrolled, 15 (56%) withdrawn/lost to follow-up. Remaining participants’ time to completion was mean 64.2, SD = 20.5 | DERS: non-significant decrease at post-treatment | Reduction,  Tunneling,  Tailoring,  Personalisation,  Self-monitoring,  Reminders,  Suggestion |
| Fonesca et al. [23] 2019, Portugal,  RCT | *Be a Mom,* CBT, 5 weeks, weekly, website access.  Technical support and reminders only as needed. | Early postpartum women with risk factors or symptoms of postpartum depression  *Be a Mom* arm: 98 allocated, 33 (34%) lost to follow-up.  Control arm: 96 allocated, 14 (14%) lost to follow-up. | DERS-SF: Significantly greater decrease in emotion regulation difficulties in intervention arm | Reduction,  Tunneling,  Tailoring,  Personalisation,  Self-monitoring,  Rehearsal,  Reminders,  Suggestion,  Similarity,  Liking |
| Bernstein et al. [29] 2022, USA,  Single-arm pre-post | *None,* CBT, 4-5 weeks, 6x daily, mobile application.  None. | Outpatients (immediately post-discharge) with suicidal ideation or behaviour  25 enrolled, 6 (24%) lost to follow-up. | ERS: Significant decrease in ERS from baseline to post-assessment | Reduction,  Tunneling,  Personalisation,  Self-monitoring,  Rehearsal,  Rewards,  Suggestion,  Similarity |
| Stappenbeck et al. [93] 2021, USA,  RCT | *None*, DBT & Social Learning Theory, 2 weeks, daily, website access.  Reminders only. | Female university students with lifetime self-reported sexual assault.  Intervention arm: 100 allocated, 5 (5%) lost to follow-up. Mean completion 10.0/14.8 (SD=4.8)  Control arm: 100 allocated, 10 (10%) lost to follow-up | DERS: significant time x condition effect with reduced emotion regulation difficulties in intervention arm | Tunneling,  Tailoring,  Personalisation, Rehearsal,  Reminders,  Suggestion, |
| Salamin et al. [77] 2019, Switzerland, open-label uncontrolled pilot | *e-motion*, DBT, 16 weeks, intended frequency not stated, website access.  Treatment support as-needed (via online forum). | Close relatives of people with mental illness.  Online intervention arm: 44 enrolled, 13 (30%) lost to follow-up  In-person intervention arm: 60 enrolled, 22 (37%) lost to follow-up | DERS: significant time x condition medium effect size on difficulties with emotion regulation | Reduction,  Tunneling,  Self-monitoring,  Simulation,  Rehearsal,  Praise,  Suggestion,  Social Role |
| Glück & Maercker [94] 2011, Germany & Austria, RCT | *None*, mindfulness, 2 weeks, 6 days per week, website access.  Technical support as-needed and reminders. | Adults (no further inclusion criteria)  Intervention arm: 28 allocated, 2 (7%) lost to follow-up, 10 (36%) completed <6 days of training  Control arm: 21 allocated, 3 (14%) lost to follow-up. | SEK-27: no significant effect on emotion regulation for time, condition, or time x condition | Reduction,  Tunneling,  Rehearsal,  Suggestion |

### Tabulation of Anger Interventions

Three studies, all RCTs, targeting symptoms of anger were identified, with publication years ranging from 2014 – 2020. A total of 655 participants were enrolled. Two of the studies were deemed to have moderate risk of bias (39, 40) and one was at high risk of bias (41). Details are reported below in Table S6.

Table S6: Study Characteristics of Anger Interventions

| **Author, Year, Location** | ***Intervention name*, modality, duration, frequency & access method.**  **Degree of Human Involvement in Treatment** | **Population, Enrollment, Attrition & Adherence** | **Outcome measure(s) & Summary** | **PSD Elements Employed** |
| --- | --- | --- | --- | --- |
| Howie & Malouff [95] 2014, USA,  RCT | *None,* CBT, 1 month, daily, website access.  Treatment support, as-needed. | Adults with elevated trait anger  CBT arm: 37 randomised, 10 (27%) lost to follow-up, 26 (70%) did not complete intervention  Control arm: 38 randomised, 6 (16%) lost to follow-up | TAS: Completer analysis found significant time x condition effects on trait anger, ITT analysis was borderline significant (*P =* 0.06) | Reduction,  Self-monitoring,  Rehearsal,  Reminders, Suggestion. |
| Johnson et al. [26] 2020,  USA,  RDICT (randomised delayed-intervention controlled trial) | *None,* relaxation therapy, 6 weeks, weekly, website access.  None. | Adults with aggression and emotion-related impulsivity.  235 randomised in total, intervention arm: 127, waitlist: 108. 121 (57%) non-response. 4.31 (out of 6) sessions completed on average. | MOAS: Significant time x condition effects on overt aggression scale. | Reduction,  Tunneling,  Self-monitoring,  Rehearsal,  Suggestion |
| Osgood et al. [96] 2020, USA,  RCT,  *Study 2 only.* | *None,* hostile bias modification training (HBMT), 1 session.  None. | Adults (no other specified characteristics).  345 were randomised but 116 (34%) were withdrawn after randomization for inadequate performance on HBMT.  Active HBMT arm: 117 completed, 8 (7%) lost to follow-up.  Sham HBMT arm: 112 completed, 4 (4%) lost to follow-up | TAS: No significant effect on trait anger | No PSD elements mentioned or apparent based on publication |

Risk of Bias Results
i. Randomised Controlled Trials:

RCTs were assessed for risk of bias using Cochrane’s ROB2 tool and visualized using the *robvis* tool (McGuinness & Higgins, 2020). Figure S1 (below) shows outcomes for each domain within the tool. There was widespread risk of bias, with only 3 (9%) of the RCTs judged to have overall low risk, 22 (69%) for which authors had some concerns, and 8 (25%) at high risk of bias. Most of this risk arose in Domain 5: Bias in selection of the reported result, in which 22 (69%) of studies were judged to have some concerns or high risk. There were some concerns about bias due to deviations from the intended interventions (10 studies, 31%) and missing data (11 studies, 34%).


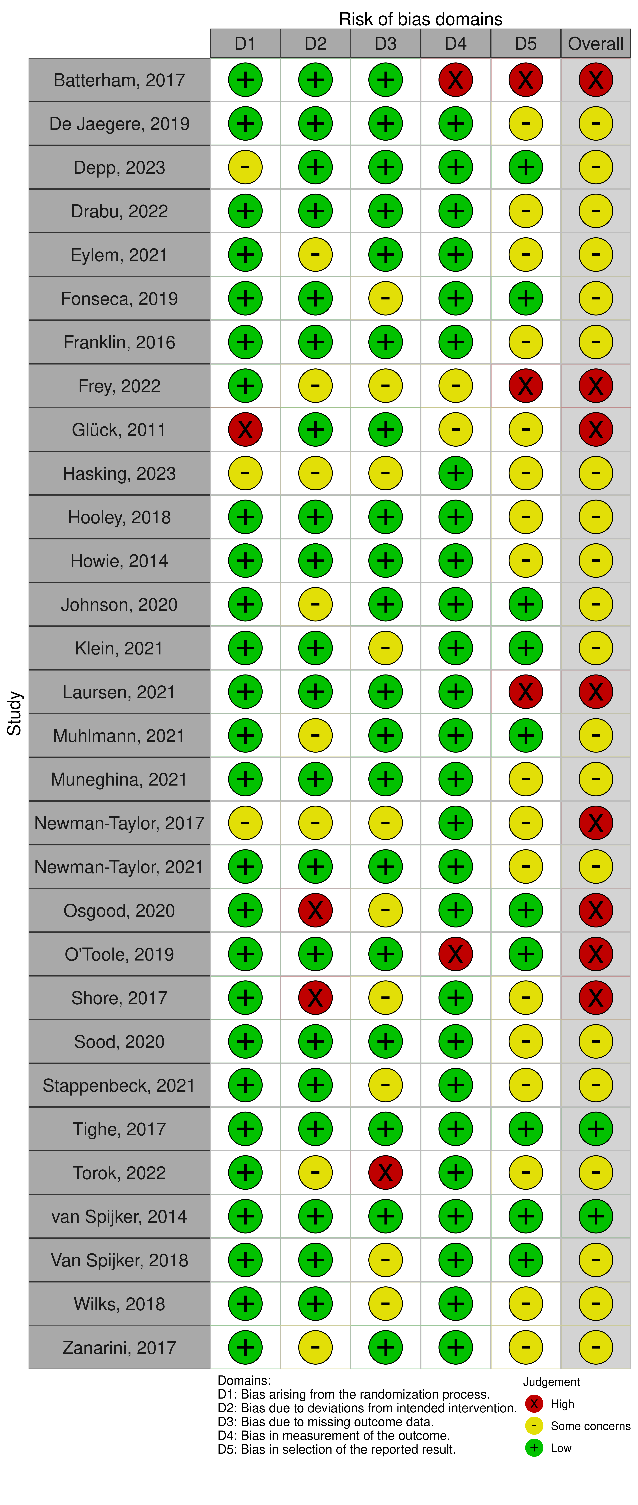


Figure S1: Risk of Bias results for RCTs, presented by bias domain.

Figure S2 shows a summary of results for each domain. Most of the risk came from Domain 5: selection of the reported result, which was due to trials not pre-registering their protocols and analysis plans.


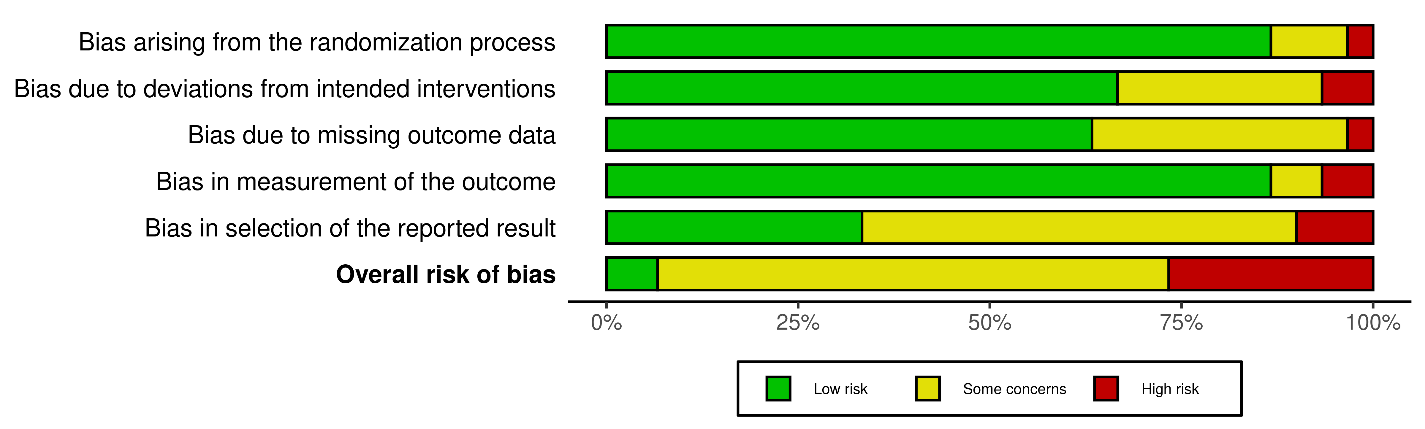


Figure S2: Risk of bias summary for each domain

### ii. Cluster trial:

There was one cluster trial included in the review, for which risk of bias was assessed using Cochrane’s ROB2 for Cluster Trials (Figure S3). We had some concerns about bias in the solitary cluster-randomised controlled trial, primarily due to the lack of a pre-registered analysis plan. Other concerns arose because it was not specified whether the randomization allocation sequence was concealed until allocation. Also, as with most studies of psychological interventions, some degree of risk arose due to participants self-reporting outcome measures without being blinded to their allocation.


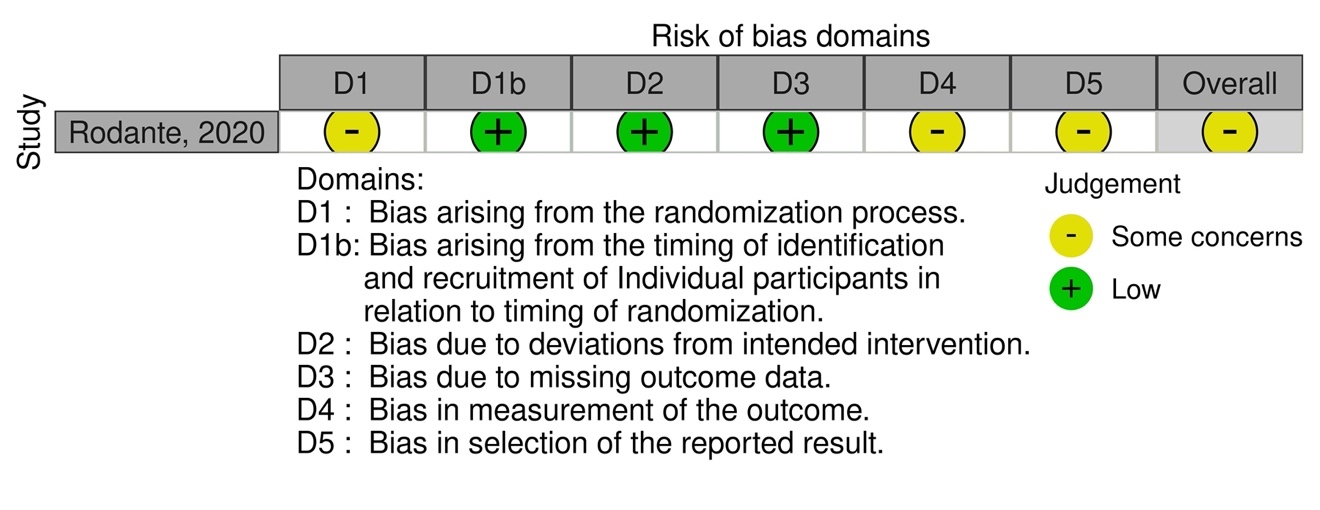


Figure S3: Risk of bias domain results for cluster trial

### iii. Single-Arm & Open-Label Studies:

Studies were assessed using the National Institute for Health’s Study Quality Assessment tool. Figure S4 below shows the outcomes for each study and domain, while Figure S5 shows the summary breakdown by domain. These studies also carried a considerable risk of bias, with just 1 (11%) deemed “Good” using the NIH Quality Assessment tool. All the studies were at risk of bias from Domain 8: Assessor Blinding, which as discussed above is difficult to avoid with psychological interventions. Most studies (8 studies, 89%) also failed each of Domain 9: Loss to Follow-Up and Domain 11, which rewards studies that took multiple baseline and follow-up measures of the primary outcome variable. We deemed Domain 11 to be overly stringent for our assessment since taking multiple baseline measures is not common practice in studies of psychological interventions.


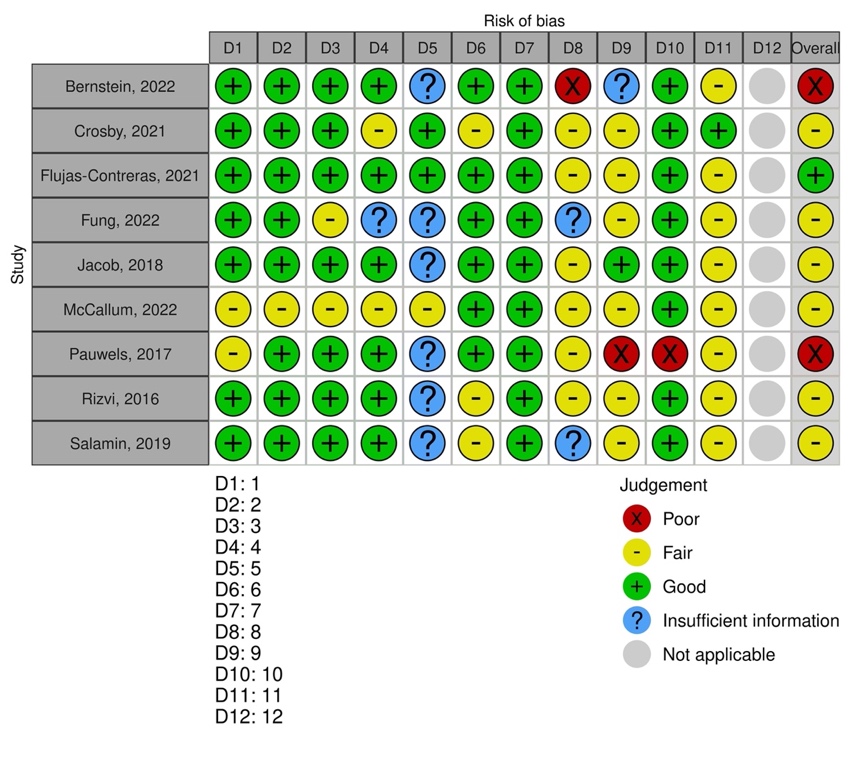


Figure S4: Risk of bias domain results for single-arm and open-label studies, by domain


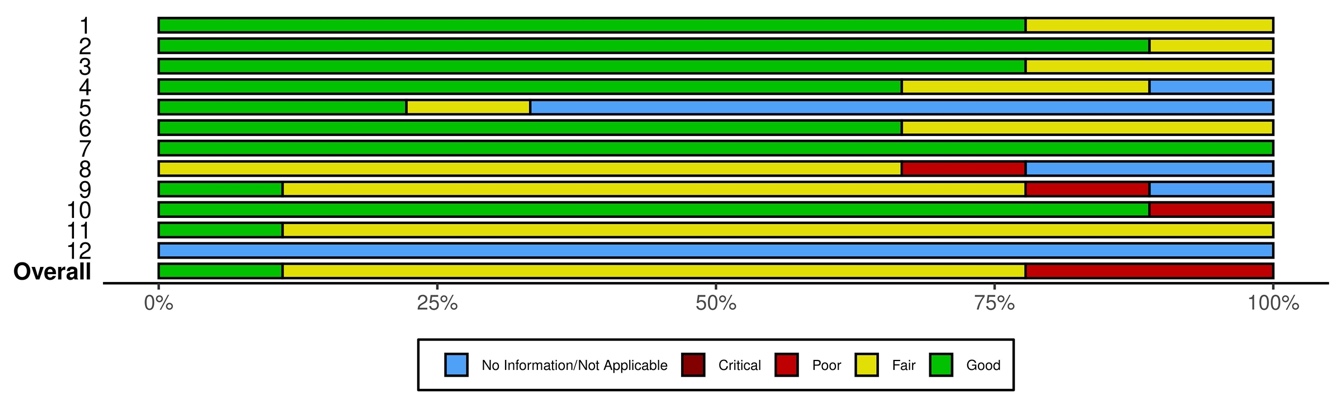


Figure S5: Summary of quality assessment results for single-arm and open-label studies, by domain

### Frequency of Persuasive System Design Elements


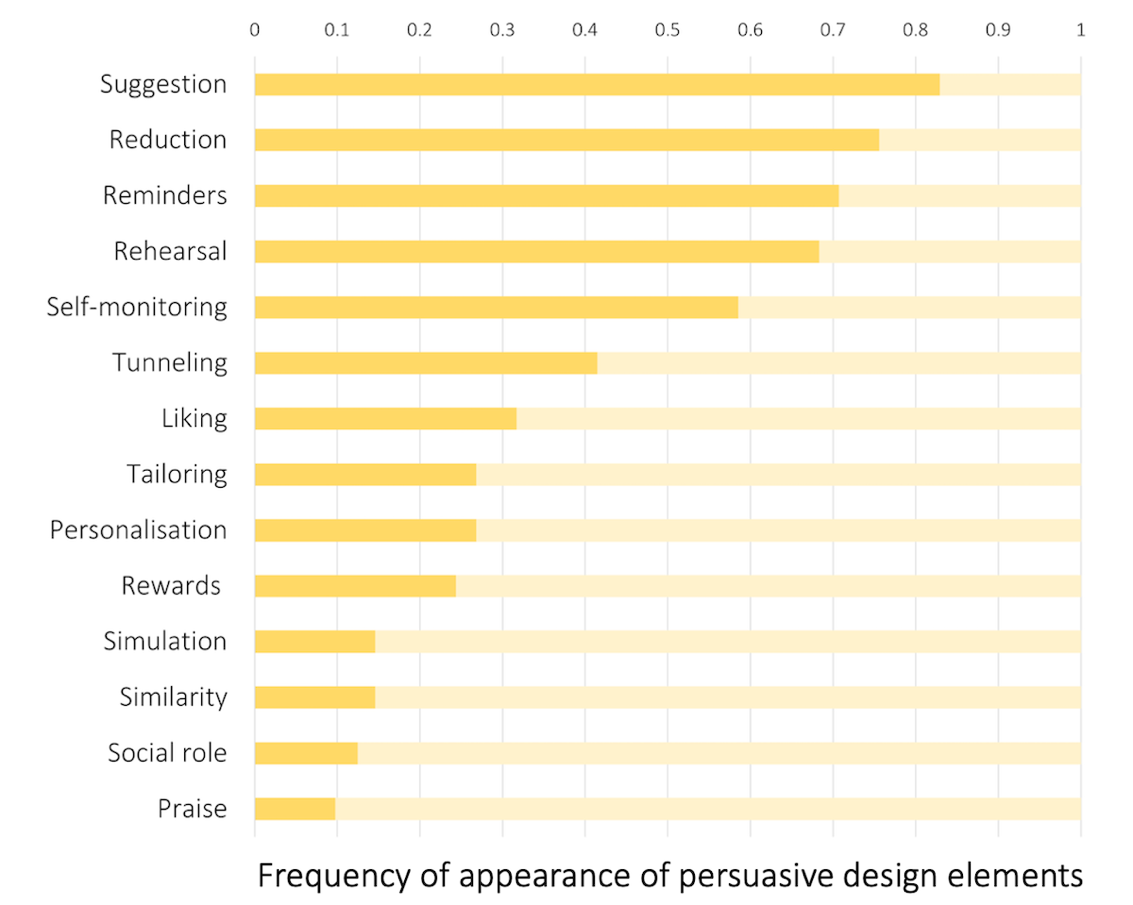


Figure S6 above shows the frequency of each persuasive system design element being employed in the included interventions.

### Meta-Analysis of NSSI RCTs

A random-effects model fitted to the data found a small, insignificant reduction in number of NSSI episodes after digital intervention: N = 5, *g* = -0.06 with 95% CI: [-0.32; 0.19], *P* = 0.52. The between-study heterogeneity variance was estimated at τ^2^ = 0.00, 95% CI: [0.00; 0.46] and between-study heterogeneity at I^2^ = 6% [0.0%; 80.5%] (Figure S7 [27, 76, 86]). Funnel plotting did not reveal evidence of publication bias (Figure S8 [27, 76, 86]).

­­
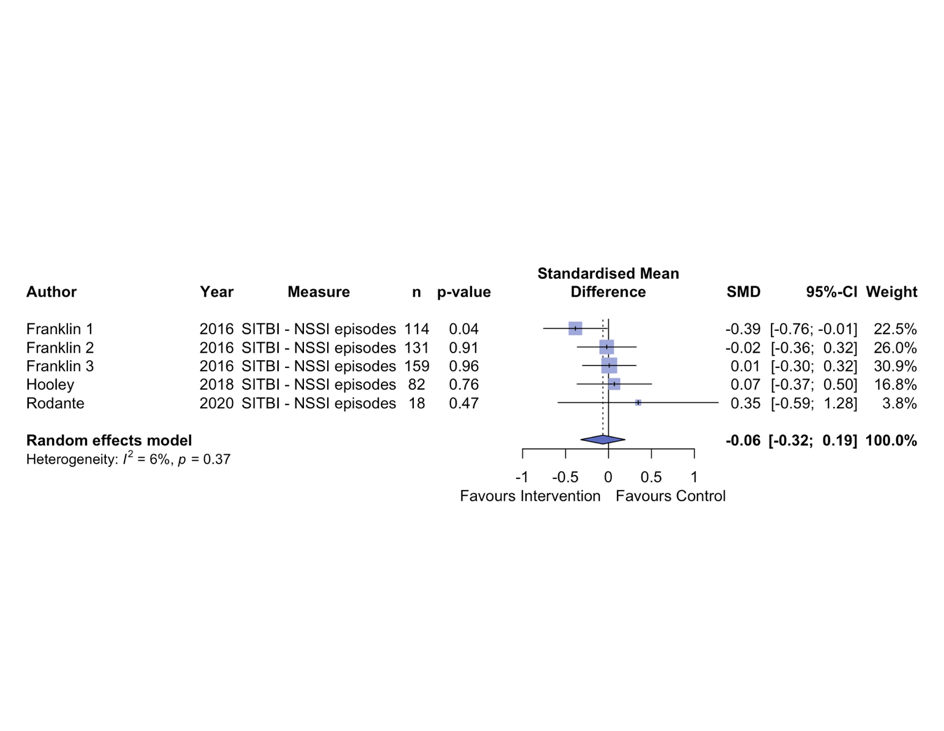


Figure S7: Forest plot of SMD in NSSI episodes after digital intervention


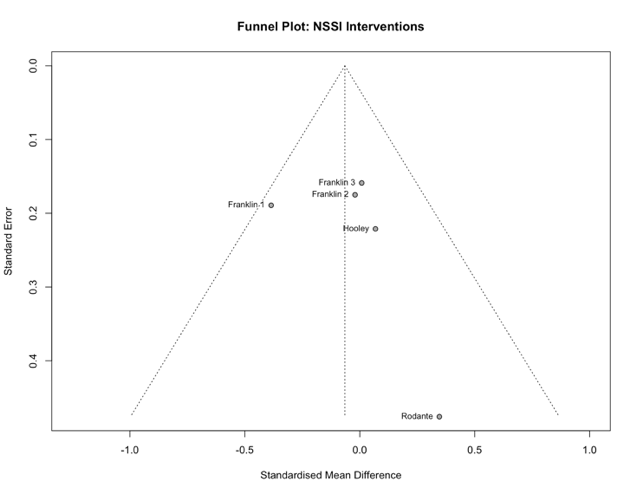


Figure S8: Funnel plot of SMD of NSSI episodes does not show risk of publication bias. Each data point represents an RCT and is labelled with the first author’s name.

### Meta-Analysis of Emotion Regulation RCTs

A random-effects model fitted to the data found a small, insignificant effect of digital interventions for emotion regulation: N = 4, *g* = -0.02 with 95% CI: [-0.55; 0.51], *P* = 0.90. The between-study heterogeneity variance was estimated at τ^2^ = 0.06, 95% CI: [0.00; 1.69] and between-study heterogeneity at I^2^ = 56.8% [0.0%; 85.7%] (Figure S9 [23, 82, 93, 94]). The funnel plot in Figure S10 [23, 82, 93, 94] does not show evidence of publication bias.


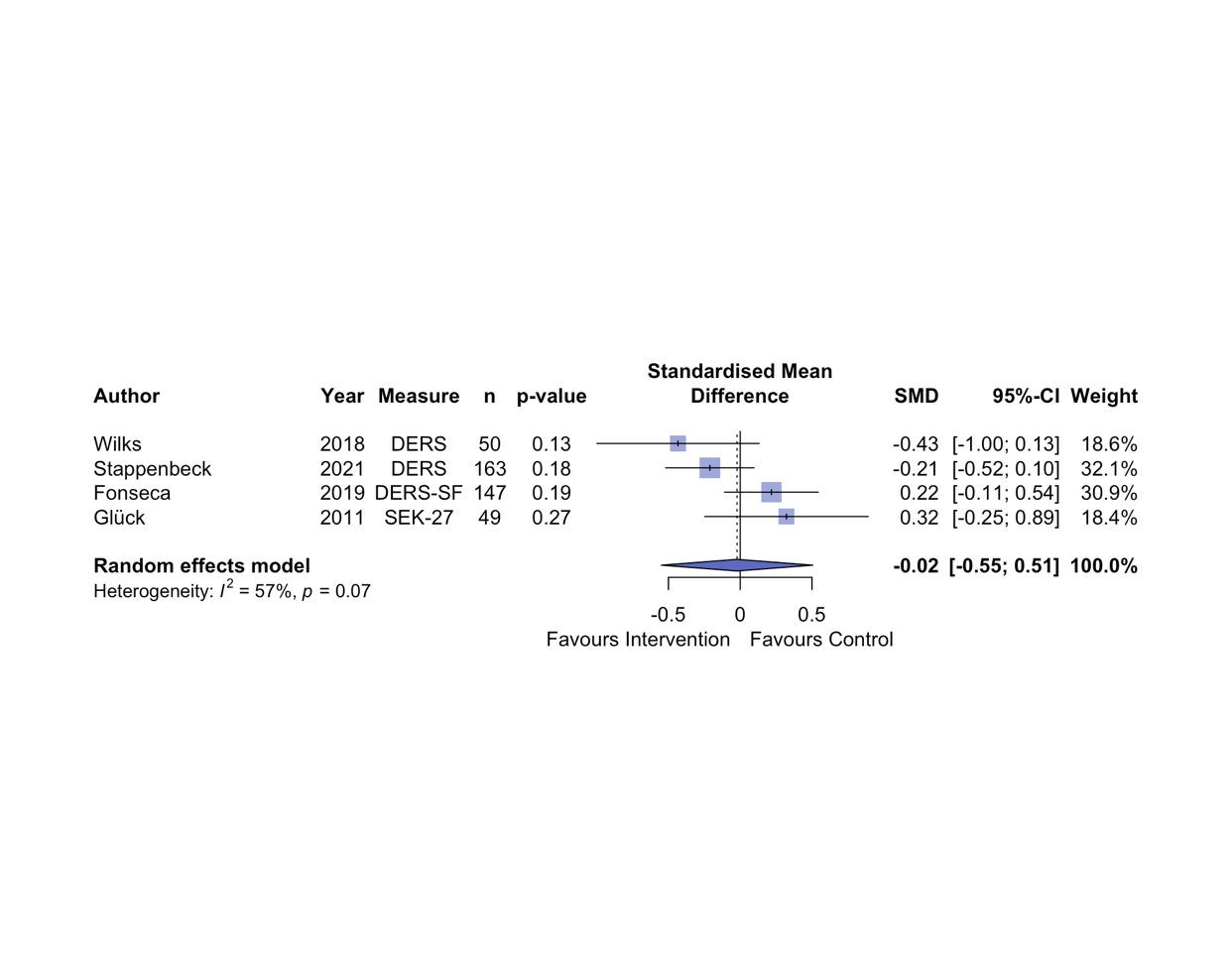


Figure S9: Forest plot of SMD in difficulties with emotion regulation. Note: the SEK-27 measures emotion regulation competencies so its SMD was reversed for inclusion in this meta-analysis

Funnel Plot of Emotion Regulation RCTs

_
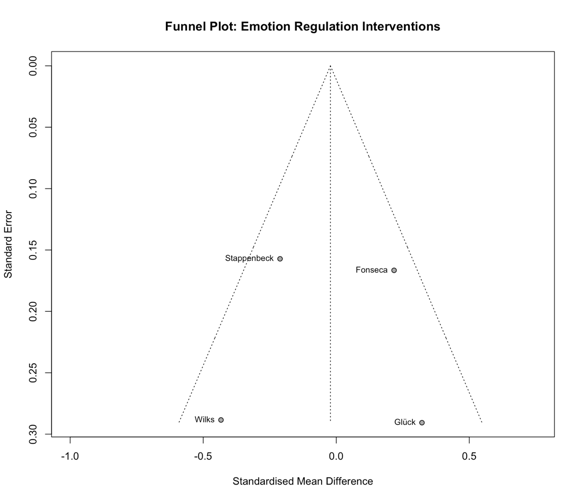
_

Figure S10: Funnel plot of effect sizes of emotion regulation interventions does not show evidence of publication bias. Each data point represents an RCT and is labelled with the first author’s name.

### Subgroup Analysis of Population in SI Interventions

Table S7 below shows that separating the studies by population (outpatient versus community-drawn samples) did not suggest significant differences in treatment effect between groups (Community SMD: -0.14, Outpatient SMD: -0.09, *p* = .77).

Table S7: Subgroup analysis comparing suicide ideation intervention studies recruiting from community versus outpatient samples

|  | *n* studies | SMD | 95% CI | *P* | *I^2^* | 95% CI | *P_subgroup_* |
| --- | --- | --- | --- | --- | --- | --- | --- |
| Population |  |  |  |  |  |  | .77 |
| Community | 13 | -0.14 | -0.28 to 0.00 | .04 | 31.1% | 50.1 – 83.8% |  |
| Outpatient | 5 | -0.09 | -0.51 to 0.32 | .56 | 71.5% | 0 – 73.6% |  |

### Meta-analysis of Anger-Targeting RCTs

A random-effects model fitted to the data found a small, insignificant effect of digital interventions for anger: N = 3, *g* = -0.17 with 95% CI: [-1.03; 0.69], *P* = 0.49. The between-study heterogeneity variance was estimated at τ^2^ = 0.10, 95% CI: [0.01; 4.43] and between-study heterogeneity at I^2^ = 80.9% [40.3%; 93.9%] (Figure S11 [26, 95, 96]). The funnel plot in Figure S12 [26, 95, 96] shows possible evidence of publication bias, but there are too few studies to conduct an Egger test.


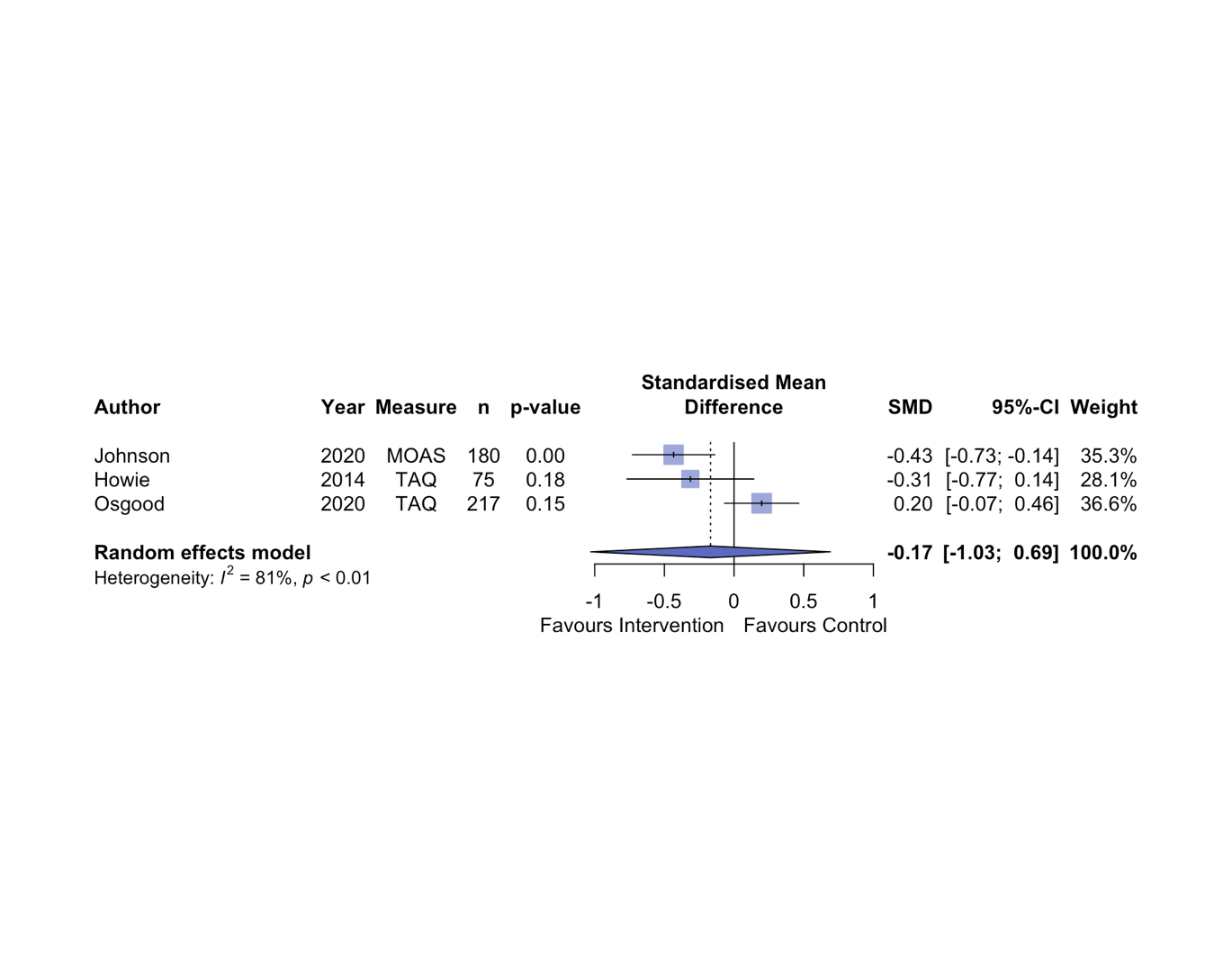


Figure S11: Forest plot of SMD of digital anger interventions

### Funnel Plot of Anger-Targeting RCTs


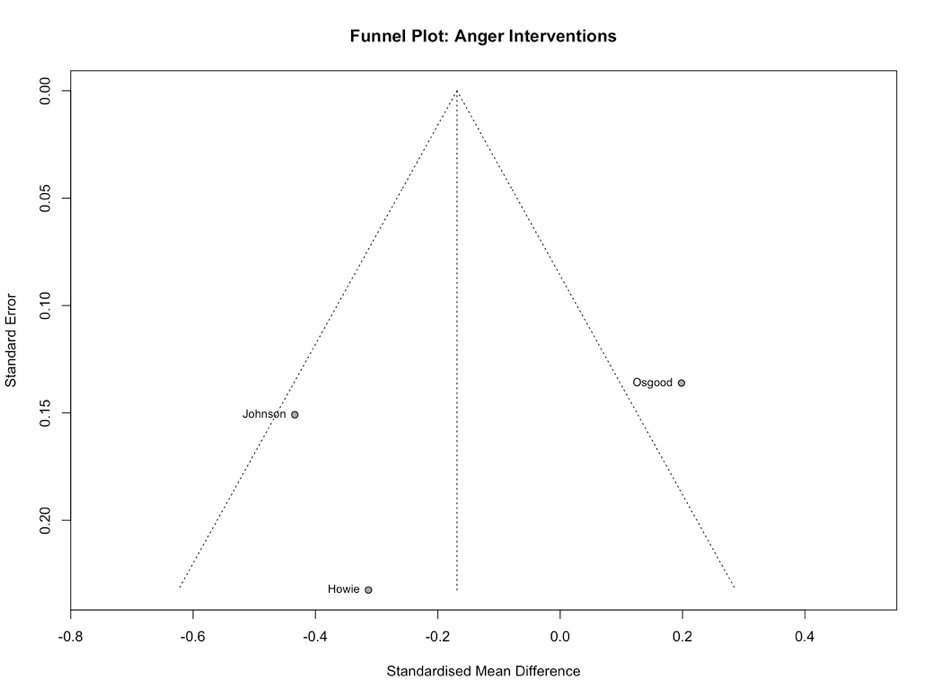


Figure S12: Funnel plot of digital interventions for anger shows possible publication bias, but there are too few studies to conduct an Egger test. Each data point represents an RCT and is labelled with the first author’s name.

# Reference List

McGuinness, LA, Higgins, JPT. Risk-of-bias VISualization (robvis): An R package and Shiny web app for visualizing risk-of-bias assessments. Res Syn Meth. 2020; 1- 7. https://doi.org/10.1002/jrsm.1411
